# A Method for High‐Throughput Screening of Monoclonal Antibody Internalization Using a DNA/Protein Molecular Staple

**DOI:** 10.1002/smtd.202401399

**Published:** 2025-09-23

**Authors:** Lara M. Mollé, Cameron H. Smyth, Bruna Rossi Herling, Daniel Yuen, Angus P.R. Johnston

**Affiliations:** ^1^ Drug Delivery, Disposition and Dynamics Monash Institute of Pharmaceutical Sciences Monash University Parkville Victoria 3800 Australia

**Keywords:** antibody internalization, flow cytometry, protein engineering

## Abstract

The use of monoclonal antibodies (mAbs) as therapeutics or for targeted delivery to specific cells is reliant on understanding mAb‐receptor interactions. Of particular importance is the relationship between mAb binding and internalization into cells. Internalization can be determined using molecular sensors; however, the challenges associated with functionalizing and purifying mAbs with these sensors often mean internalization is not assessed. To address this, we have developed a simple, one‐pot approach that requires no chemical modifications of the mAbs that efficiently quantifies mAb binding and internalization into cells. This is achieved by developing a protein staple that binds a DNA based internalization sensor to mAbs. This sensor is used to screen mAb binding and internalization into T cell and B cell receptors present on human peripheral blood mononuclear cells. The differences between cell binding and receptor internalization can be rapidly assessed for an array of lymphocyte receptors. These results demonstrate that high binding does not necessarily correlate to high uptake into the cell or high internalization efficiency of a receptor. Receptor internalization efficiency is a product of intrinsic characteristics of both the receptor and mAb, highlighting the need to screen for these functions to improve mAb therapeutics.

## Graphical abstract

Internalization of antibodies plays a critical role in targeted drug delivery and the activity of antibody therapeutics. A simple, one‐pot, chemical modification free approach is developed to quantify antibody internalization into cells.

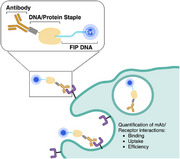

## 1 Introduction

The ability to target therapeutics to specific cells based on the presence of cell surface proteins is key to the field of precision medicine.^[^ ^1^, ^2^ ^]^ Antibodies can be used for targeting purposes, by functionalizing nanoparticles with antibodies to deliver larger and more complex therapeutic payloads.^[^ ^3^ ^]^ This has been recently implemented using antibody targeted LNPs carrying an mRNA cargo for hematopoietic stem cell modification.^[^ ^4^ ^]^ Antibodies can also be used as stand‐alone therapies. Therapeutic monoclonal antibodies (mAbs) can directly block cell surface receptor functions^[^ ^5^ ^]^ and simultaneously mark target cells for destruction by antibody dependent cell‐mediated cytotoxicity.^[^ ^6^ ^]^ Antibodies can also be used to target therapeutics to specific cells, such as CD22 selective antibody‐drug conjugate Inotuzumab,^[^ ^7^ ^]^ which can be used to deliver cytotoxic payloads.

A key factor in the activity of these antibody therapeutics is understanding how rapidly and efficiently their target receptors are internalized into cells. Many factors contribute toward the internalization kinetics of an antibody, including binding epitope and affinity^[^ ^8^ ^]^ and target receptor biology.^[^ ^9^ ^]^ For many antibody conjugates, internalization is a critical step to allow access to intracellular sites of drug activity. Conversely, many antibody therapeutics ideally persist at the cell surface and function best when not internalized. Antibody therapies such as Rituximab^[^ ^5^, ^10^ ^]^ block or modulate cell‐cell interactions, while others such as Mosunetuzumab (bispecific anti‐CD20 and anti‐CD3) bridge immune cell types to facilitate immunomodulation.^[^ ^11^ ^]^

Despite the therapeutic value of selecting an antibody for optimal internalization or surface persistence, measurement of this activity is often overlooked due to the complexity of accurately quantifying uptake. Standard flow cytometry measurements measure cellular association, but cannot distinguish between surface‐bound or internalized material.^[^ ^12^ ^]^ Several techniques have been developed to evaluate internalization of antibodies. These include image analysis,^[^ ^13^ ^]^ surface washing or stripping,^[^ ^14^ ^]^ secondary antibodies,^[^ ^15^ ^]^ and sensor‐based approaches.^[^ ^16^, ^17^, ^18^ ^]^ Image‐based analysis typically has low throughput and is inherently subjective. Surface wash or stripping is limited by the efficiency of washing/stripping, resistance to protease treatments, and compromised cell viabilities.^[^ ^12^ ^]^

An alternative strategy is implementing secondary antibodies to help quantify the internalization of a primary fluorescently labeled antibody of interest.^[^ ^12^ ^]^ This involves using a fluorescent primary antibody that binds to its receptor to promote internalization. If a fluorescent secondary antibody is added, it binds to the primary antibody of interest if it remains on the cell surface. This distinguishes surface‐bound material (double positive for both the primary antibody and the secondary antibody) from internalized material (single positive for only the primary antibody). Quantitative measurements using this method can be challenging due to varying antibody off‐rates and overlapping fluorophores,^[^ ^15^ ^]^ limiting assay throughput and the complexity of samples that can be investigated.

**Figure 1:**
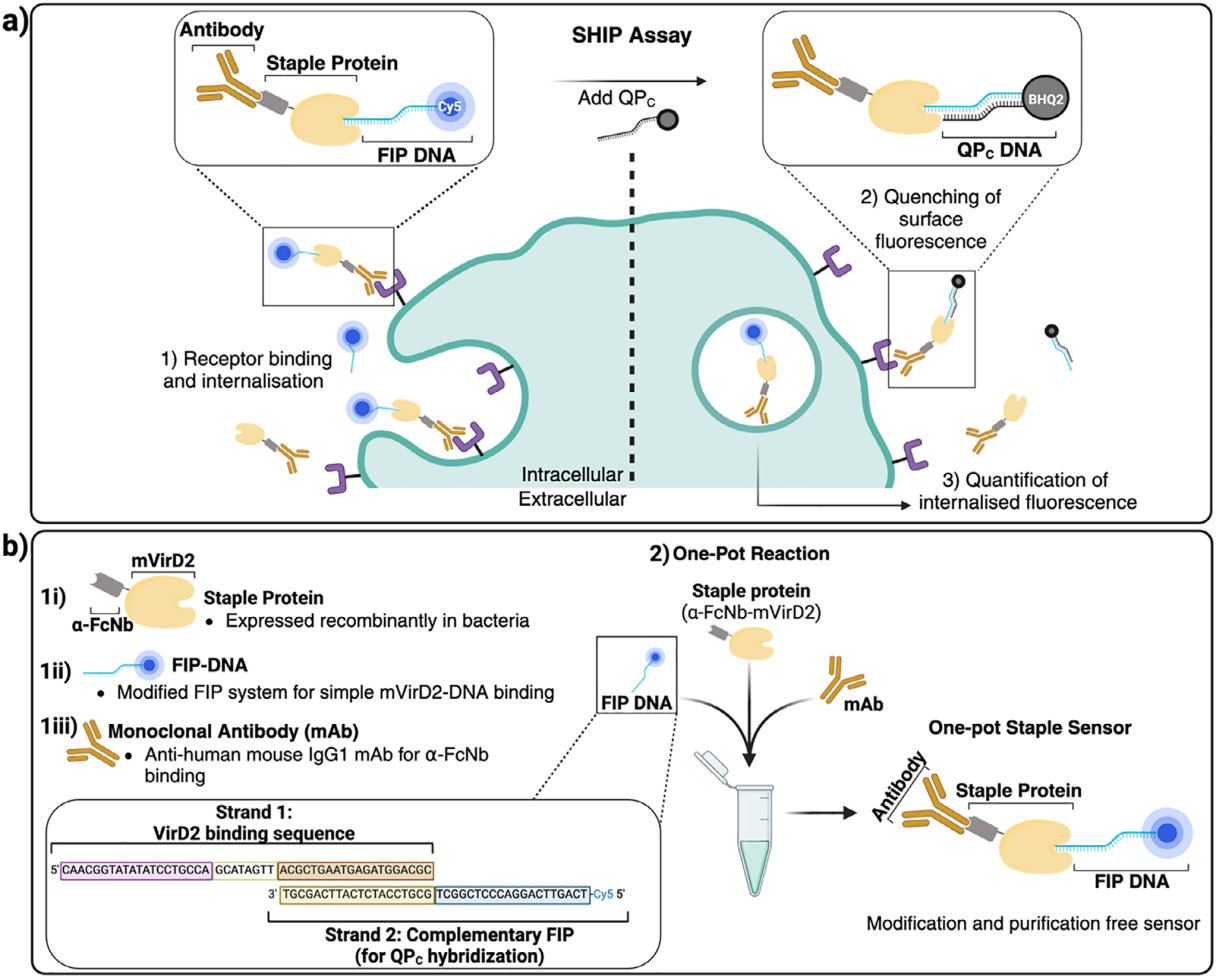
The one‐pot staple sensor used for high‐throughput screening of antibody binding and internalization. a) The SHIP assay using the SHIP staple sensor. Quantifying cellular internalization requires 1) sensor incubation with cells to promote internalization. 2) Surface signal quenching. 3) Flow cytometry to measure sensor signal. b) Staple sensor assembly only requires two steps: i) production of recombinant staple, and acquisition of ii) the FIP‐DNA and iii) mAb of interest from commercial sources, 2) combining all components in a 1:1:1 ratio in a single tube. The staple sensor self‐assembles and can be directly used for uptake and receptor internalization assay.

Alternative methods distinguish between cell surface and internalized signal, using a quenching component. Quenchable antibody systems, which employ fluorescence‐quencher pairs,^[^ ^15^, ^19^ ^]^ enable the detection of internalized signal in the absence of surface signal, allowing for accurate quantification of antibody internalization. These quencher‐based assays enable high throughput (not limited to single cell) screening using flow cytometry and can be used in more complex, heterogenous cell populations. Previously, we have demonstrated a specific hybridization internalization probe (SHIP) to quantify protein internalization into cells using flow cytometry using a quencher‐based approach.^[^ ^20^, ^21^, ^22^ ^]^ The assay enables the internalization of materials functionalized with the fluorescent internalization probe (FIP) to be quantified (**Figure** **1**). FIP, a single stranded DNA oligonucleotide functionalized with a Cyanine‐5 dye (Cy5), can be conjugated to the antibody of interest. This can subsequently be quenched at the cell surface to determine the amount of unquenched, internalized sensor, allowing the calculation of receptor internalization efficiency.

While the SHIP assay,^[^ ^20^, ^22^ ^]^ similarly to other quencher based approaches, offers simple and quantitative measurement of antibody internalization, it involves the chemical conjugation of the FIP DNA (or other assay dependent fluorophores^[^ ^15^, ^19^ ^]^) to the antibody, which requires a chemically modified oligonucleotide, chemical modification of the antibody and multiple time‐consuming purification steps (Figure S1, Supporting Information). This dramatically limits the throughput of antibodies that can be screened for their internalization properties. There is a need for a simple, conjugation and purification free method to assess the internalization of antibodies.

Herein, we have developed a simple bioconjugation system that allows conjugation of a fluorescently labelled DNA biosensor to antibodies without modification to the antibody or DNA and does not require complex and time‐consuming purification steps (Figure 1b). We have achieved this by developing a one‐pot protein staple that acts as a bridge between antibodies and DNA. Implementing this one‐pot staple sensor system enables numerous mAb‐receptor interactions to be rapidly characterized in parallel. We have demonstrated the utility of this system by using it to attach our SHIP sensor to 13 different T and B cell specific antibodies. This high throughput screen of mAb/receptor interactions demonstrated that mAb uptake and receptor internalization efficiency is dictated by total cell association as well as intrinsic receptor internalization properties.

## 2 Results and Discussion

### 2.1 Development of One‐Pot Staple Sensor

To quantify receptor internalization using the SHIP assay, attachment of a fluorescent internalization probe (FIP) to an antibody of interest is required. Previously, we used an N‐hydroxysuccinimidyl ester‐dibenzocyclooctyne (NHS‐DBCO) linker to generate a DBCO‐modified antibody (Figure S1, Supporting Information). A strain‐promoted alkyne‐azide cycloaddition is subsequently used to attach the FIP, a DNA oligonucleotide synthesized with 5′ Cy5 and 3′ azide moieties (Figure S1, Supporting Information). Each step requires purification to remove unreacted reagents, which adds to the complexity of the process. This conjugation method is robust but results in random functionalization of amine residues (using succinimidyl ester chemistry) which has the potential to occur within the Fab region and negatively affect binding of the antibody to its antigen.^[^ ^23^ ^]^ As such, employing conjugation methods that ensure attachment within the Fc region are preferable.^[^ ^24^, ^25^ ^]^

To simplify this attachment process, we designed a novel fusion protein composed of a modified *Agrobacterium tumefaciens* DNA‐binding protein Virulence factor D2 (mVirD2)^[^ ^26^, ^27^ ^]^ and an anti‐Fc nanobody (anti‐FcNb).^[^ ^24^ ^]^ In this DNA/protein staple, the mVirD2 selectively binds and forms a covalent bond to oligonucleotides containing a specific sequence (5′ CAACGGTATATATCCTGCCA 3′), while anti‐FcNb binds with high affinity and specificity to mouse IgG1 antibodies. The anti‐FcNb binds specifically to the conserved fragment crystallizable (Fc) region of the mouse IgG1 antibody.^[^ ^24^ ^]^ Recent work using cryo‐electron microscopy has shown that the binding domain is at the base of the Fc region and therefore is unlikely to inactivate the fragment antigen binding (Fab) region.^[^ ^28^ ^]^ By attaching the sensor to the Fc domain, the Fab domain is free to bind to the target receptor, which can mediate receptor specific internalization. Furthermore, since the Fc region for an antibody isotype within a species is conserved, the anti‐FcNb can bind any IgG1 antibodies raised in a mouse. Here, mouse IgG1 antibodies raised against human receptors (anti‐human) were used to generate the staple sensor. Therefore, mixing the DNA/protein staple, antibody and FIP DNA that contains VirD2 binding sequence results in spontaneous “stapling” together of the components, without the need for chemical modification or purification steps (Figure 1b).

To demonstrate that the DNA/protein staple can capture mAbs, the DNA/protein staple was incubated with a mouse IgG1 antibody at an equimolar ratio for 60 min at 37 °C. After separation of the mixture using SDS‐PAGE, immunoblotting indicated that the DNA/protein staple bound >98% of the mAb (Figure S2, Supporting Information). Similarly, incubating the DNA/protein staple with a 1:1 molar ratio of FIP‐DNA resulted in ≈33% binding of the FIP‐DNA. This is consistent with previous reports of mVirD2‐DNA binding efficiency to cognate recognition sequences at a 1:1 molar ratio.^[^ ^26^ ^]^ This ratio of DNA/protein staple: DNA: mAb (1:1:1 molar) was maintained for all further experiments.

**Figure 2:**
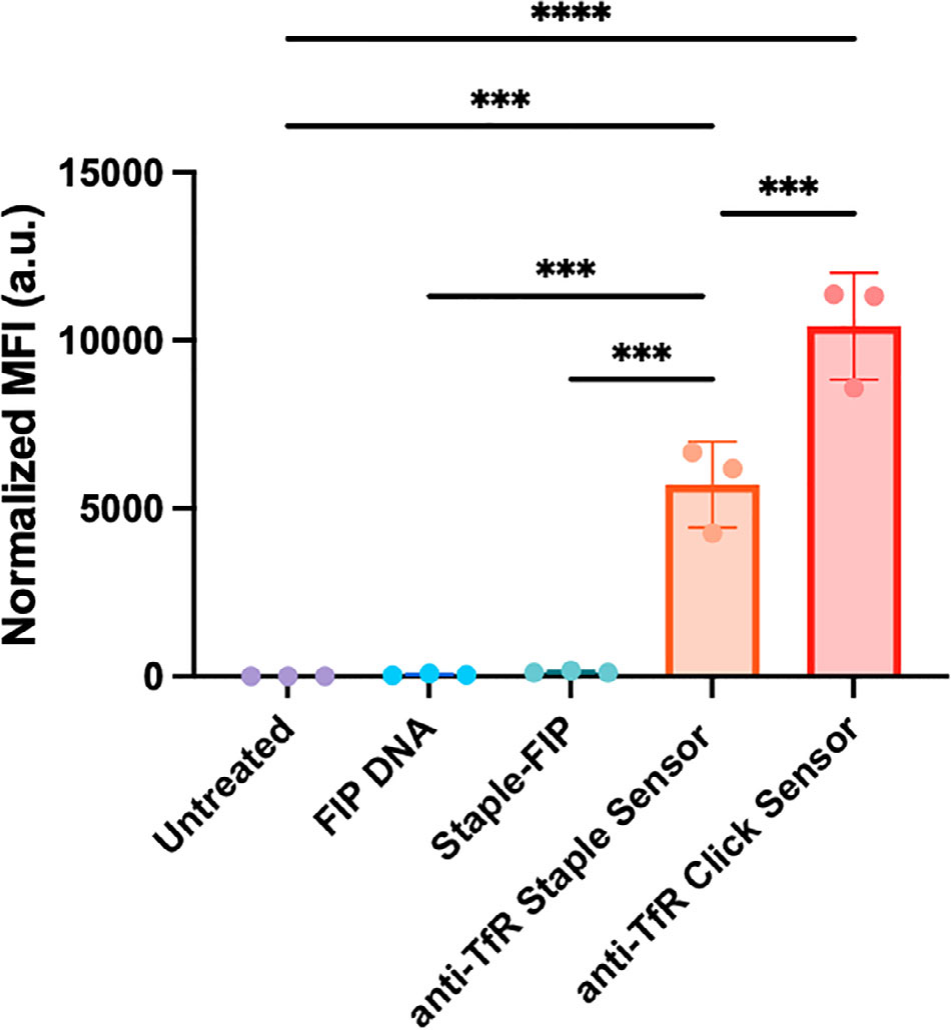
Self‐assembled staple sensor binds to TfR on cultured B cells. One‐pot anti‐TfR staple sensor TfR was incubated with B‐lymphoblastoid (C1R) cells for 1 h at 37 °C. MFI (Cy5) was measured by flow cytometry. The fluorescent signal for each sample was normalized for DOL. Ordinary One‐Way ANOVA, *p* > 0.05 is not significant and is not indicated on the graphs. *** = *p* < 0.001 and **** = *p* < 0.0001, mean ± SD (*n* = 3). Each data point represents the mean of one experiment (three technical replicates).

To confirm the assembled staple sensor retained antibody activity, we selected transferrin receptor (TfR) as a model for studying receptor binding and internalization, as it has high surface expression and rapid membrane turnover.^[^ ^29^ ^]^ The one‐pot human anti‐TfR staple sensor (*staple sensor*) was compared to the click sensor, the same sensor system prepared using click chemistry (**Figure** **2**; Figure S3, Supporting Information). Using the anti‐TfR mAb (clone *OKT9*) and C1R (B‐lymphoblastoid) cells, binding was confirmed using flow cytometry. The staple sensor and click sensor were added to cells at a final concentration of 25 nM of anti‐TfR SHIP sensor (Figure 2; Figure S4, Supporting Information). To assess if uncoupled FIP‐DNA or Staple‐FIP non‐specifically binds to the cells, both reagents were added at three times the concentration (75 nM) of the assembled sensor. Cells were then analyzed by flow cytometry. The click sensor (MFI 10418.7 ± 1593.5) showed higher signal than staple sensor (MFI 5701.0 ± 1274.4). This results in the one‐pot sensor having slightly lower sensitivity than the click sensor. However, the signal is sufficient to quantify both association and internalization. The anti‐TfR staple sensor showed significantly higher cell association compared to untreated cells (MFI 4.3 ± 0.7) and significantly higher cell binding than the FIP‐DNA (MFI 49.2 ± 27.5) and staple‐FIP (MFI 130.9 ± 38.0) controls. This demonstrates the FIP‐DNA only associates with the cells when it is bound to the antibody. To confirm free FIP‐DNA has minimal impact on the signal, we incubated cells with 30x (2250 nM) the concentration of free sensor (Figure S6, Supporting Information) and observed minimal nonspecific cell uptake of the FIP‐DNA or staple‐FIP.

### 2.2 One‐Pot Quantification of Receptor Internalization In Vitro

After demonstrating that the anti‐TfR staple sensor bound to TfR on cultured cells, we investigated the staple sensor's ability to evaluate internalization. Hybridization and quenching of the surface Cy5 signal using the quenching DNA probe (QP_C_) was conducted at 4 °C. This process prevents internalization of the antibody/receptor complex, ensuring all bound antibody remains on the surface, allowing quenching efficiency to be quantified. As our original FIP oligonucleotide sequence is conserved in this system, our previously described complementary quenching probe (QP_C_) sequence functions identically in this system. Both staple and click sensors reached >96% quenching, confirming that cell‐surface signal from both sensors is efficiently quenched by QP_C_ (bearing a 3′ BHQ2) (Figure S7, Supporting Information). Receptor internalization efficiency (%) is determined by ratioing the internalized fluorescence signal (after the quencher has been added) to the total cell association of the sensor. This is a relative measure of receptor internalization and is normalized to receptor abundance.

**Figure 3:**
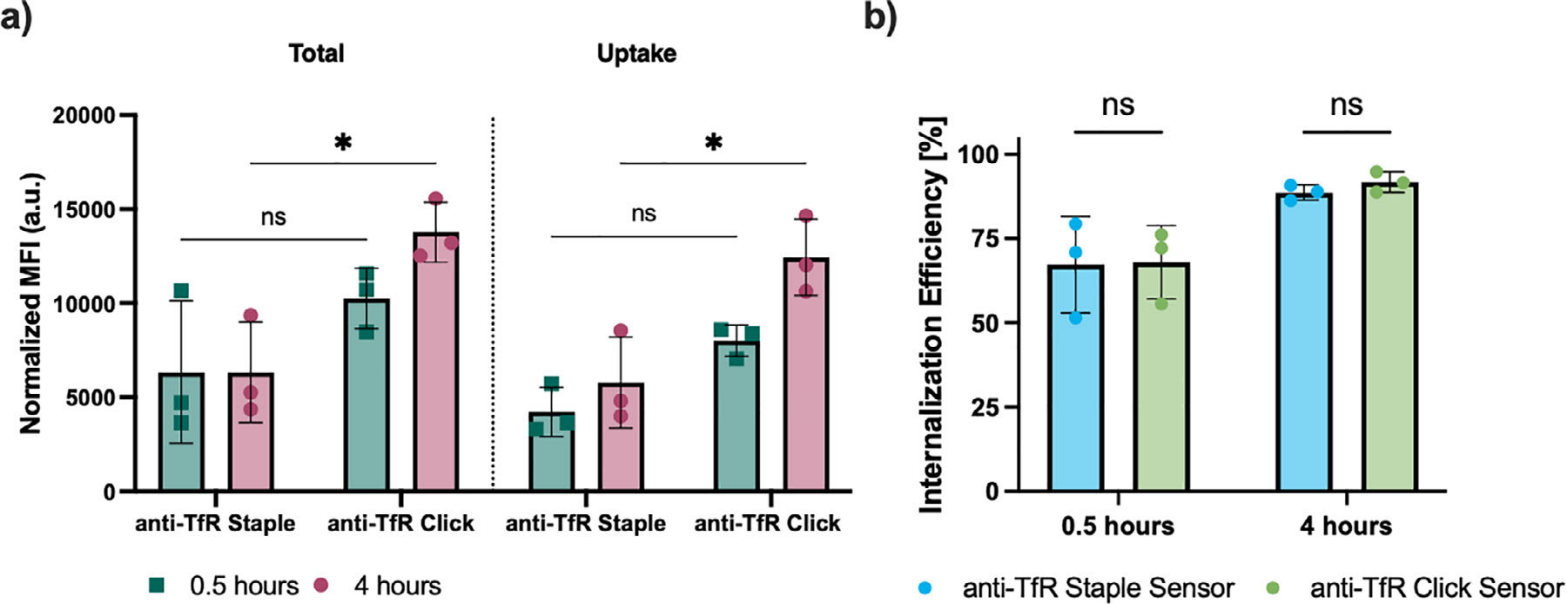
The one‐pot staple system exhibits equivalent performance to the click sensor. a) Cell association (MFI) and b) percentage internalization of the click and staple sensors at 0.5 and 4 h in C1R cells, measured using flow cytometry. Sensor was used at 25 nM, quencher was used at 500 nM. The fluorescent signal for each sample was normalized for DOL. Statistical analysis was performed using unpaired T‐test. For all data: *p* > 0.05 is not significant and is not indicated on the graphs. * = *p* < 0.05, mean ± SD (*n* = 3). Each data point represents the mean of one experiment (three technical replicates).

Here, we confirmed that differences in the total cell signal did not impact the ability of the one‐pot staple sensor to accurately measure the receptor internalization efficiency (**Figure** **3**a). Using both methods, we observed rapid internalization of TfR, with ≈65% internalization measured by both methods after 30 min (Figure 3b). After 4 h, both methods showed ≈90% internalization. These results agree with previous reports from us and others that measured the uptake kinetics of TfR.^[^ ^21^, ^30^, ^31^ ^]^

These data demonstrate that the novel DNA/protein staple used to construct the staple sensor does not hinder the receptor internalization measurements by the sensor. This validates the DNA/protein staple as a feasible substitute for click chemistry to construct the SHIP internalization sensor. The simplicity of this method is amenable to automation, as only rudimentary liquid handling is required. Parallelizing sensor attachment would allow panels of compatible mouse IgG1 antibodies to be prepared and used to investigate receptor expression levels, uptake levels of materials, and internalization efficiency (%) of a receptor.

### 2.3 Rapid Screen of Binding, Uptake, and Receptor Internalization of Multiple Antibodies in Lymphocytes

Targeting T cells and B cells is an established strategy in immunotherapy. Antibodies are implemented for T and B cell depletion to effectively treat several lymphomas and autoimmune disorders,^[^ ^2^, ^32^ ^]^ however improved therapies are required for treatment resistant cancers. Furthermore, using antibodies to target nucleic acid‐based nanotherapeutics such as siRNA and mRNA nanoparticles for immunomodulation is rapidly evolving, making these specialized cell types of particular interest as targets for such therapies.

Antibody therapies focus on the antigen‐specific delivery of cargo or antibody‐mediated activity, which relies on the presence of target membrane surface receptors. These cell receptors can be cell‐specific or expressed across multiple cell populations with varying abundance. Understanding mAb/receptor interactions and ensuring the mAb is interacting with its target receptor in the desired manner is essential for therapeutic outcome (**Table** **1**) and can only be achieved by assessing these interactions by measuring cell binding, uptake and receptor internalization efficiency. Each mAb/receptor interaction is unique and different receptor/antibody paired exhibit significantly different internalisation behaviour, therefore developing antibody therapies against any receptor targets, new or existing, requires an in‐depth understanding of this behavior. As such, using a method that can measure and quantify both the amount of antibody that enters the cell after binding (uptake) and how efficient this process is through the target receptor (internalization efficiency) can aid and improve the development process.

**Table 1:** Clinically relevant immune cell receptors for screening of receptor internalization in peripheral blood mononuclear cells.

Target receptor	Predominant expression	Role	Existing mAb therapeutics against the target	Refs.
CD2	T cells Some B cells	Involved in costimulatory T cell activation	*Siplizumab* *(treatment of transplant rejection)* – binding of mAb promotes T cell depletion	[33]
CD3	T cells	Responsible for T cell signaling/ activation upon MHC binding	*Otelixizumab* *(Type 1 diabetes treatment mAb)* binds and blocks the CD3 receptor to prevent T cell attack on insulin‐producing beta cells	[34]
CD4	T cells (CD4^+^)	Adhesion receptor – Stabilizes the interaction between CD4+ T cell and antigen‐presenting cell (APC)	*Engineered Anti‐CD4 mAbs* *(anti‐arthritis)* bind to CD4 (different epitopes) to suppress arthritis in rat models.	[35, 36]
CD5	T cells	Inhibits TCR/CD3 downstream signaling following T cell activation	*UCHT‐2 & L17F12 (two Anti‐CD5 mAbs)* *(anti‐cancer)* work to prevent internalization of CD5 to block inhibitory signaling improving cytotoxic effects	[37]
CD7	T cells	T cell activation after binding of endogenous ligand	*Anti‐CD7 Deruxtecan drug conjugate (in mice)* *(anti‐tumor)* targets T lymphocytes for internalization to deliver cytotoxic drug	[38]
CD8a	T cells (CD8^+^)	Adhesion receptor – Stabilizes the interaction between CD8+ T cells and APC	*Anti‐CD8 mAb (in rats)* *(autoimmune disease treatment)* acts to deplete CD8+ T cells.	[39]
CD19	B cells	Part of a signal transduction complex that activates BCR for B‐cell activation	*Tafasitamab* *(anti‐cancer mAb)* binding causes cross‐linking of the extracellular domain, leading to apoptosis. *Loncastuximab tesirine* *(anti‐tumor ADC)* mAb binds and internalizes CD19, the drug, and inhibits the DNA repair mechanism.	[40, 41, 42]
CD20	B cells	Plays a role in BCR signaling – proposed calcium flux regulation	*Rituximab* *(anti‐cancer mAb)* binds to CD20, causing redistribution into lipid rafts, and induces cytotoxicity *Tositumomab* *(anti‐cancer ADC with radioactive cargo)* is internalized to deliver a radioactive payload for cell death	[5, 10, 43, 44, 45, 46]
CD22	B cells	Inhibition of BCR signaling	*Inotuzumab‐ozogamicin* *(anti‐cancer ADC)* binding and internalization allow release of the anti‐cancer drug for DNA degradation and cell death *Epratuzumab* *(treatment of lupus erythematosus)* induces internalization of CD22 to further disrupt BCR signaling and trigger cell death.	[7, 47, 48]
CD27	T cells Some B cells	Co‐stimulatory activation of cell types	*Varlilumab* *(anti‐cancer)* stimulates the CD27 pathway for T cell activation and effector cell recruitment for antitumor activity.	[49]
CD38	B cells Some T cells	Receptor/ enzyme involved in regulating signaling pathways	*Daratumumab* *(anti‐cancer)* binds and maintains membrane‐bound CD38, acting to recruit effector cells for death or promote mAb cytotoxicity	[50]
CD44	T cells B cells	Binding and recognition of Hyaluronan	*Engineered Anti‐CD44 mAbs* *(anti‐tumor)* found to promote tumor targeting and uptake in in vivo mouse models	[51]
CD45	T cells B cells	Stabilization of extracellularly bound receptor interactions	*Anti‐CD45 ADC (in mice)* *(transplant rejection treatment)* acts to target to deliver a cytotoxic drug for depletion of host immune cells	[52]
				

Here, we demonstrate the use of the staple sensor for high‐throughput screening of antibody uptake and receptor internalization efficiency using antibodies that target a range of clinically relevant receptors on human primary T and B cells. The chosen receptors have previously been targets of therapeutic antibodies with differing internalization profiles relevant to their therapeutic mechanism (Table 1). Here, validated, commercial anti‐human mAbs against these various T and B lymphocyte cell surface receptors (Figures S8 and S9, Supporting Information), were used to demonstrate a high throughput internalization screening method on human peripheral blood mononuclear cells (PBMCs).

Antibody‐receptor internalization was investigated after 30 min and 4 h incubation with PBMCs isolated from healthy donors. One‐pot staple SHIP sensors were generated for all 13 mAbs targeting T cell and B cell receptors and were directly incubated with PBMCs for both time points, after which phenotyping antibodies were added. The association and receptor uptake were analyzed using flow cytometry, with cell subpopulations separated using phenotyping antibodies (Figure S10, Supporting Information). Cell association, uptake and receptor internalization efficiency of the SHIP staple sensors were calculated as outlined in the experimental details.

It is important to note that, as the internalization efficiency is a ratio of uptake to cell association, antibodies with very low levels of association can lead to a large amount of error (through division by a small number). Therefore, we determined a threshold for calculating the internalization efficiency of a receptor, whereby the association of the mAb must be significantly higher (*p* < 0.05) than the signal generated by the mIgG1 isotype control. MFI values less than the isotype control suggest that the mAb is interacting non‐specifically with the cells, rather than binding their target receptor.

#### 2.3.1 Targeting Therapeutic T Cell Receptors

**Figure 4:**
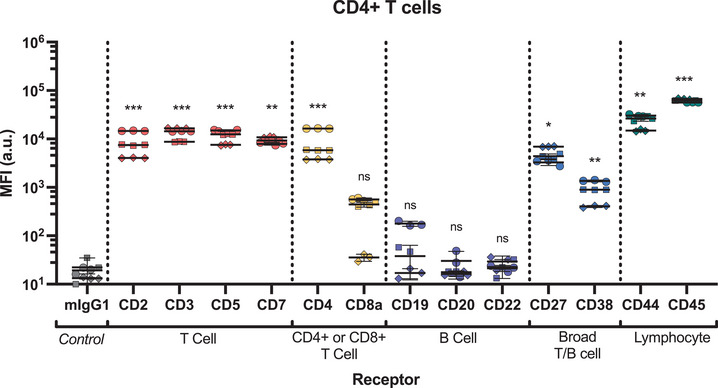
Association of a library of antibodies to human CD4+ T cells assessed using a staple sensor. Cy5 fluorescence was measured by flow cytometry. Statistical analysis was performed using 2‐way ANOVA with Dunnett's test, *n* = 3. Each mAb staple sensor was compared to the isotype (mIgG1) control. *p* > 0.05 = not significant (ns), * = *p* < 0.05, ** = *p* < 0.01, *** = *p* < 0.001 and **** = *p *< 0.0001. Significance shown is representative of the mean calculated p value of the donors. Each symbol is representative of a single donor (with technical replicates).

There is a range of T cell receptors that have been identified as clinically relevant targets and have been successfully exploited for targeted mAb‐based therapies (Table 1). As expected, the T cell receptors CD2, CD3, CD5, and CD7 were expressed at similar levels in both CD4^+^ and CD8^+^ T cell subsets (**Figure** **4**; Figures S11 and S13, Supporting Information). No significant association was observed for B cell specific receptors on either T cell subset, therefore these receptors did not have receptor internalization efficiency calculated. We also explored broadly expressed T/B cell markers (CD27 and CD38), as well as general lymphocyte makers (CD44 and CD45), which were all detected at levels significantly higher than the mIgG1 control. The association trend for all the receptors we tested was similar at both 30 min (Figure S11, Supporting Information) and 4 h (Figure 4; Figure S13, Supporting Information).

**Figure 5:**
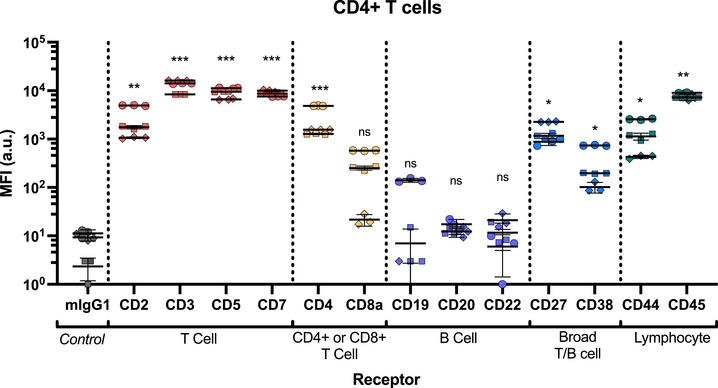
mAb uptake into CD4+ T cells after 4 h incubation. The MFI of the mAb‐staple sensor through each receptor was measured following the addition of quencher DNA (500 nM). Cy5 fluorescence was analyzed by flow cytometry. Statistical analysis was performed using 2‐way ANOVA with Dunnett's test, *n* = 3. Each mAb staple sensor was compared to the isotype (mIgG1) control. *p* > 0.05 = not significant (ns), * = *p *< 0.05, ** = *p* < 0.01, *** = *p* < 0.001. Significance shown is representative of the mean calculated p value of the donors. Each symbol is representative of a single donor (with technical replicates).

**Figure 6:**
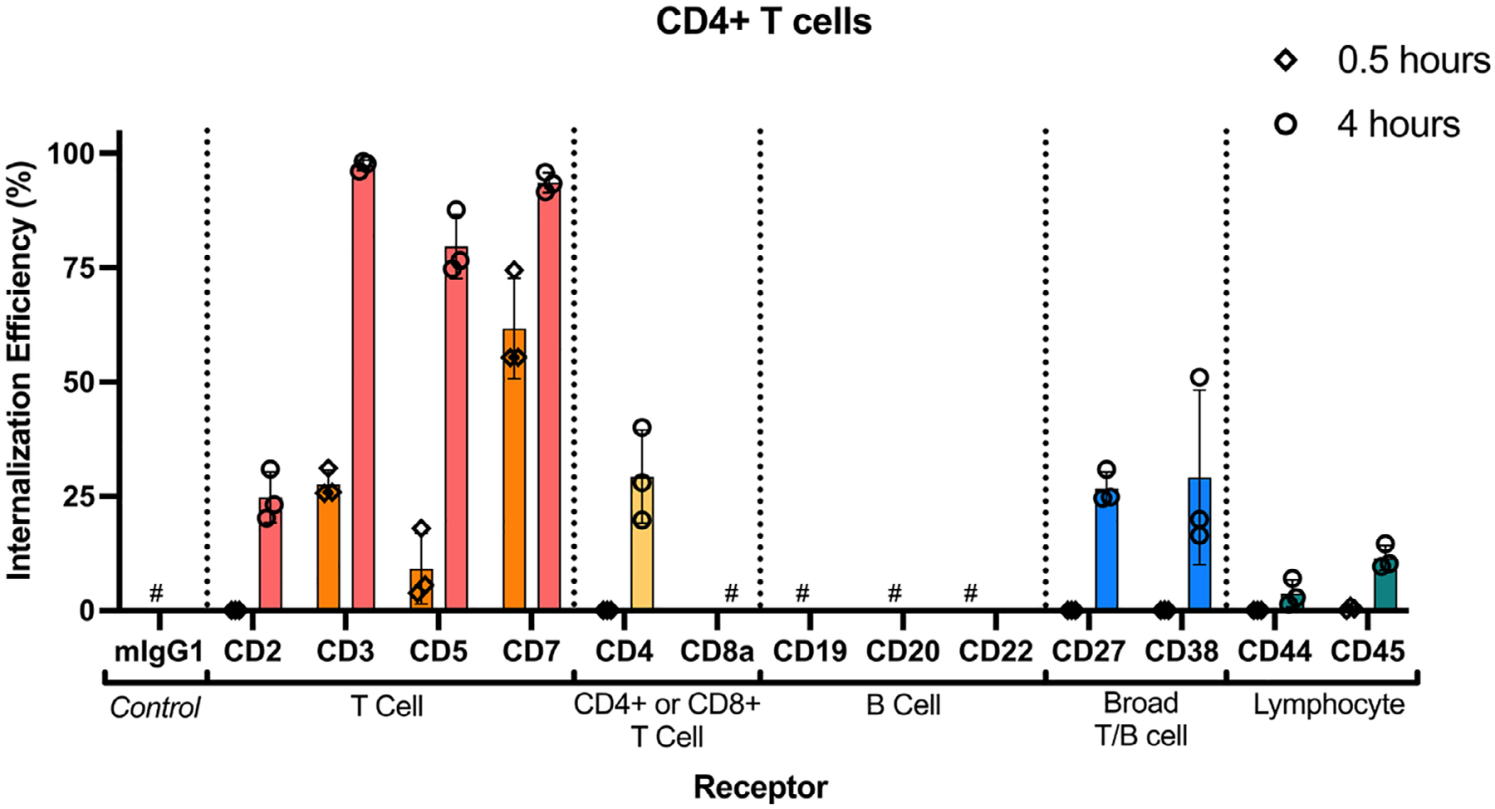
Internalization efficiency increases over time for CD4+ T cell receptors. Internalization efficiency (%) was calculated for each receptor relevant to CD4+ T cells at 0.5 and 4 h. # signifies receptors that did not have sufficient association compared to mIgG1 isotype control for internalization to be calculated. Each data point represents mean of a single donor (three technical replicates). Data is mean ± SD (*n* = 3).

In T cells, CD2 is a co‐stimulatory receptor that acts to transduce the T cell receptor (TCR)/CD3 complex, facilitating receptor signaling.^[^ ^53^ ^]^ This is thought to occur upon receptor activation, whereby CD2 intracellularly co‐localizes with other signaling molecules. Interestingly, despite relatively high expression levels on CD4+ and CD8+ T cells (MFI 8743 ± 4691 and 11259 ± 7802, respectively), uptake of antibody bound to CD2 in both T cell subsets was significantly lower than for CD3, CD5 and CD7 receptors (**Figure** **5**; Figures S12 and S14, Supporting Information). These trends are reflected in internalization efficiency, with no CD2 internalization detected after 30 min, and ≈25% and ≈20% in CD4+ and CD8+ T cells after 4 h, respectively (**Figure** **6**; Figure S15, Supporting Information). These data suggest that for this mAb clone, CD2 receptor internalization is independent of mAb binding.

In contrast, CD3 showed rapid internalization, with ≈20% of antibody internalized after 30 min and >90% after 4 h. CD3 forms part of the TCR/CD3 complex, directly responsible for T cell activation. Following TCR binding to peptides presented by MHC molecules, CD3 transmits activation signals to the T cell triggering downstream regulation of cell survival, cytokine production, proliferation, and differentiation.^[^ ^54^ ^]^ mAb uptake through CD3 is rapid and highly efficient, showing the highest total uptake (CD4 T cell MFI 12921 ± 3495 and CD8 T cell MFI 14397 ± 4021) of all T cell receptors (Figure 5; Figure S14, Supporting Information). CD3 is a highly endocytic receptor on T cells shown to be efficiently internalized into cells upon activation,^[^ ^55^ ^]^ a finding which is consistent with its rapid and high internalization efficiency seen following antibody‐mediated binding. This indicates that upon binding, this anti‐CD3 mAb activates CD3 causing rapid uptake of the mAb into the cell.

CD7 showed even more receptor internalization, reaching ≈60% internalization in CD4+ T cells and ≈40% internalization in CD8+ T cells after only 30 min. The CD7 receptor is suggested to play a co‐stimulatory role in T cell activation and promote proliferation following binding of endogenous ligands.^[^ ^56^ ^]^ Although internalization efficiency of the CD7 receptor is twice the level than that of CD3 at 0.5 h, the amount of anti‐CD3 mAb uptake is equal to that of anti‐CD7 mAb uptake (Figure S12, Supporting Information) despite the total association of anti‐CD7 being lower than that of anti‐CD3. This highlights an important point that when selecting the optimal receptor to target, the total amount of material internalized into the cell is a product of both the internalization efficiency and association to the cell.

CD5 also plays important roles in downstream T cell responses following endogenous activation of TCR/CD3 complex.^[^ ^56^, ^57^ ^]^ It has also been described as a regulator of T cell signaling following activation of the TCR via inhibitory signaling.^[^ ^57^ ^]^ Total cell association of the anti‐CD5 mAb to CD4+ and CD8+ T cells (MFI 11664 ± 3304 and 13912 ± 5027, respectively) shows it is expressed at similar levels to CD3 (Figure 4). However, anti‐CD5 mAb uptake is slower and less efficient than CD3 (Figure 5; Figure S14, Supporting Information). Only ≈9% of CD5 was internalized after 30 min (Figure 6; Figure S15, Supporting Information), however this increases to >70% after 4 h.

CD4 and CD8a receptors are functional receptors that distinguish the functions of the two major T cell subsets. Although CD4 and CD8a are classified as co‐receptors to the TCR/CD3 complex, they are adhesion molecules that help support binding and stabilization of the TCR to MHC molecules for improved antigen recognition.^[^ ^58^ ^]^ This means that whilst these receptors have a functional role in T cell activation, this role may not be reliant on internalization of these receptors.^[^ ^59^ ^]^ Our results support this, with <1% internalization of anti‐CD4 and anti‐CD8a mAb through their respective receptors after 30 min (Figure 6; Figures S12 and S15, Supporting Information). After 4 h, internalization increased to ≈30% of the bound antibody, which was similar to the uptake observed for anti‐CD2.

Pan T and B cell markers were also investigated to understand whether mAb binding and internalization profiles vary between cell types. CD27 is proposed to play a co‐stimulatory role in the activation of cells.^[^ ^60^ ^]^ CD38 is a receptor and enzyme involved in regulating signaling pathways related to cell activation, proliferation, and differentiation in both cell types.^[^ ^61^, ^62^ ^]^

Both CD27 (CD4 T cell MFI 4875 ± 1667 and CD8 T cell MFI 4500 ± 838) and CD38 (MFI 885 ± 414 and 408 ± 255) expression on T cells is lower than expression of the T cell specific markers. No uptake or internalization was detected for either receptor after 30 min, with ≈30% after 4 h (Figure 6; Figures S12 and S15 (Supporting Information).

We also investigated the behavior of common lymphocyte receptors, CD44 and CD45, which are highly abundant on CD4^+^ and CD8^+^ T cells (Figure 4; Figure S13, Supporting Information).^[^ ^63^, ^64^ ^]^ CD44 primarily plays a role in the binding and processing of hyaluronan,^[^ ^65^, ^66^, ^67^ ^]^ but has also been implicated as an adhesion protein to stabilize T cell interactions with other immune cells at the cell membrane.^[^ ^63^, ^68^, ^69^ ^]^ Similarly, CD45 is primarily tasked with extracellularly stabilizing other membrane bound receptors (e.g., TCR) to aid in activation.^[^ ^70^ ^]^ While these receptors are highly abundant on T cells (CD44, MFI >20000 and CD45, MFI >60 000; Figure 4; Figure S13, Supporting Information), receptor internalization efficiency was low. <1% internalization was observed after 30 min, and CD44 receptor internalization reached ≈4% after 4 h, while CD45 reached ≈10% after 4 h (Figure 6; Figure S15, Supporting Information). The high expression level of CD45 means that despite the inefficient internalization, the total amount of antibody uptake was greater than CD2 and similar to CD5 and CD7. This again highlights the importance of understanding both receptor internalization efficiency and total mAb uptake.

#### 2.3.2 Therapeutic B Cell Markers

Like T cells, B cells and B cell‐specific receptors have also been implicated in different cancers and other diseases, therefore have been targets for clinical mAb therapies (Table 1). Other receptors however may provide internalization routes for payload delivery for new therapeutic interventions.

**Figure 7:**
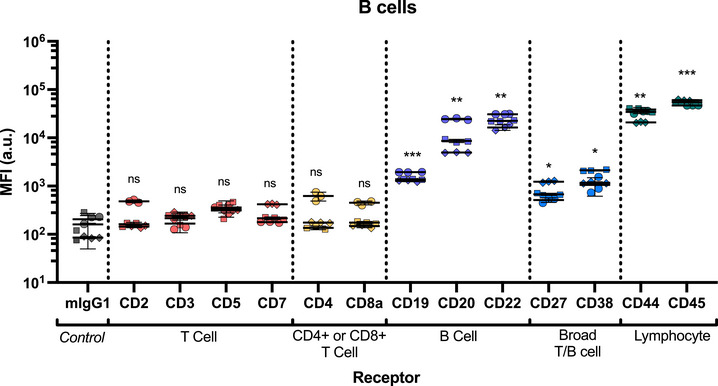
Ab total cell binding/ association in B cells demonstrates relative expression levels of receptors after 4 h. Association of each mAb with each receptor was measured using the mAb‐staple sensor. Cy5 fluorescence was analyzed by flow cytometry at 649 nm to detect Cy5‐FIP. Statistical analysis was performed using 2‐way ANOVA with Dunnett's test, *n* = 3. Each mAb staple sensor was compared to the isotype (mIgG1) control. *p* > 0.05 is not significant (ns), * = *p* < 0.05, ** = *p* < 0.01, *** = *p* < 0.001. Significance shown is representative of the mean calculated p value of the donors. Each symbol is representative of a single donor (with technical replicates).

Non‐specific association of mIgG1 to B cells (MFI 151 ± 79) was higher than for T cells (CD4 T cells MFI 18 ± 8 and CD8 T cells MFI 13 ± 9), but still significantly lower than association of the B cell‐specific markers (**Figure** **7**). As expected, T cell specific antibodies showed the same level of non‐specific association as the mIgG1 isotype control (Figure 7; Figure S11, Supporting Information). Therefore, these receptors were therefore excluded from calculations of receptor internalization efficiency.

**Figure 8:**
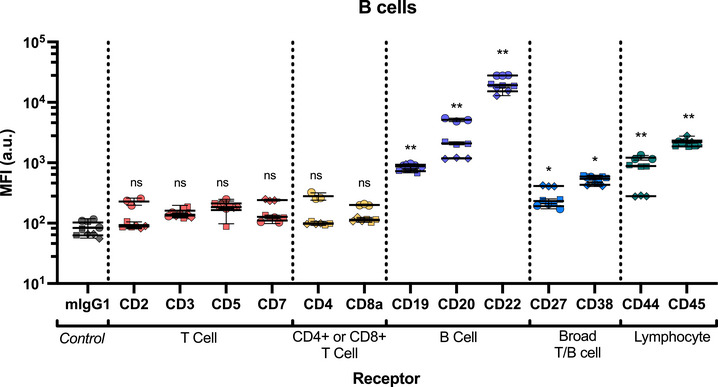
mAb uptake into B cells after 4 h incubation. The MFI of the mAb‐staple sensor through each receptor was measured following the addition of quencher DNA (500 nM). Cy5 fluorescence was analyzed by flow cytometry at 649 nm to detect Cy5‐FIP. Statistical analysis was performed using 2‐way ANOVA with Dunnett's test. Each mAb staple sensor was compared to the isotype (mIgG1) control. *p* > 0.05 is not significant (ns), * indicates *p* < 0.05, ** indicates *p* < 0.01. Significance shown is representative of the mean calculated p value of the donors. Each symbol is representative of a single donor (with technical replicates). Data is *n* = 3.

First, we focused on three B‐cell‐specific receptors, CD19, CD20, and CD22. The CD19 receptor plays a major role in B cell activation, acting as a co‐receptor responsible for aiding in B cell responses to T cell‐dependent antigens.^[^ ^71^ ^]^ CD19 works synergistically with the B cell receptor (BCR) to reduce the activation threshold for B cell activation, enhancing B cell specificity and sensitivity toward antigens.^[^ ^72^ ^]^ Of the three B‐cell‐specific markers tested, CD19 shows the lowest expression levels (CD19 MFI 1537 ± 309, CD20 MFI 12697 ± 9017, CD22 MFI 23312 ± 6415; Figure 7). While no internalization was detected after 30 min, ≈50% of CD19 was internalized after 4 h. This however does not equate to high mAb uptake (**Figure** **8**), due to the low expression levels of CD19.

In contrast, CD20 exhibited ≈8x times higher association, but only ≈17% internalization after 4 h. CD20 mAb therapies, such as Rituximab used to treat Non‐Hodgkin lymphoma via cancerous B cell depletion,^[^ ^5^ ^]^ are important examples which highlight the need to understand mAb uptake and effect of receptor internalization. Some CD20 mAb therapies require the mAb to remain surface‐bound to induce cytotoxicity,^[^ ^5^ ^]^ whilst others function by internalization.^[^ ^46^ ^]^ The role of CD20 remains poorly understood, but it has been proposed that it could interact directly with the BCR or may also function as a calcium channel involved in B cell activation.^[^ ^46^, ^73^ ^]^ There was significant variation in the CD20 levels from different donors, but interestingly, the internalization efficiency was similar in all three donors. Some CD20 mAbs have been proposed to require co‐receptors (like FcyRs) for internalization which are recruited upon ligation.^[^ ^46^ ^]^ This may explain the high levels of cellular association without the corresponding internalization.^[^ ^46^ ^]^

In contrast to CD19 and CD20, CD22 shows more efficient uptake.^[^ ^74^ ^]^ CD22 is proposed to play multiple roles in B cell immune responses, being linked to inhibition of BCR signaling^[^ ^75^ ^]^ and showing high expression (MFI 23312 ± 6415; Figure 7). The receptor internalization kinetics were similar to CD3 on T cells, with ≈35% internalization after 30 min and ≈89% after 4 h. Previous work has identified that CD22 internalizes specifically upon mAb binding,^[^ ^74^, ^76^, ^77^ ^]^ which is consistent with our data here.

**Figure 9:**
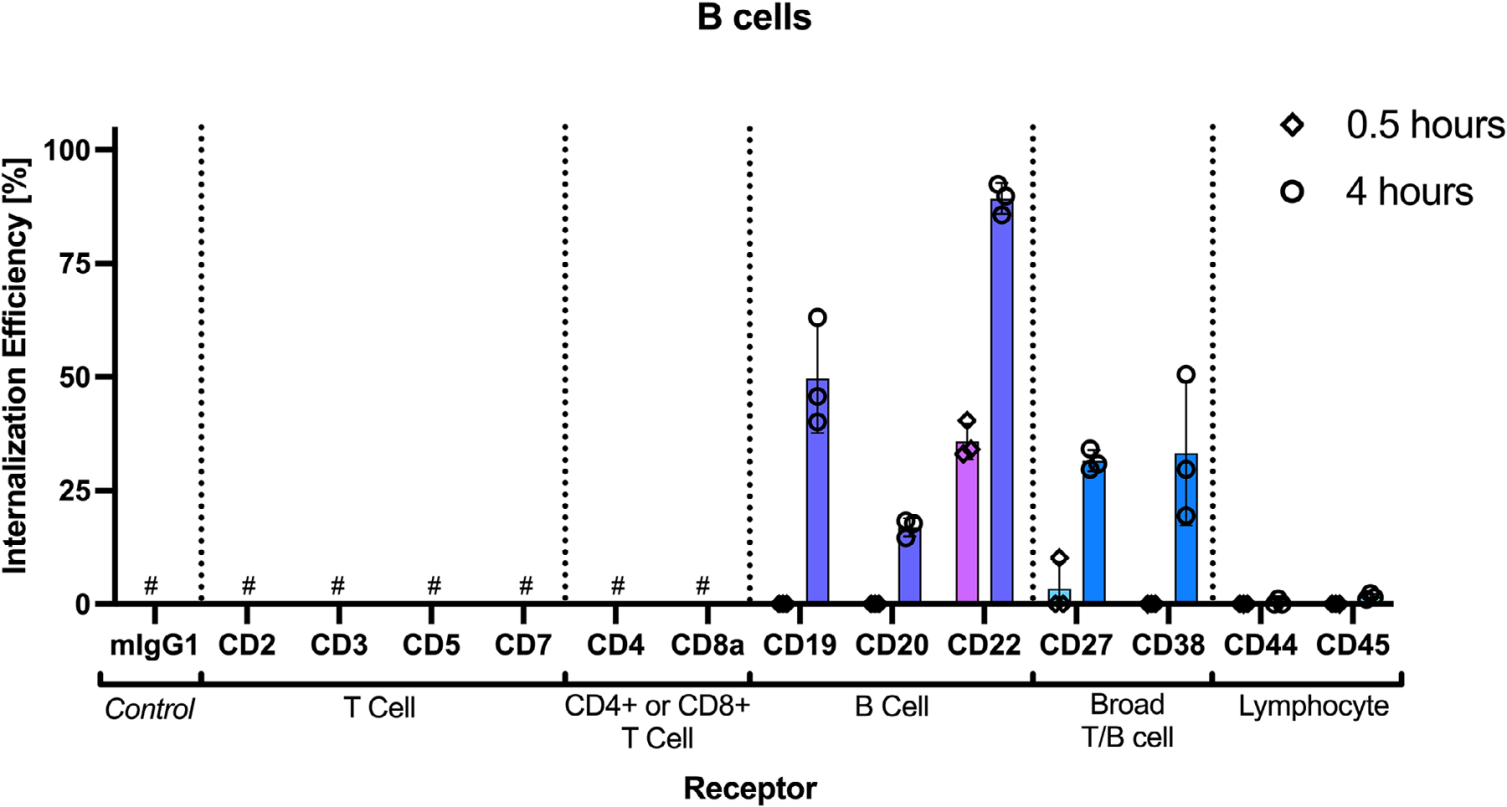
CD22 is rapidly internalized on B cells. Internalization efficiency (%) was calculated for each receptor at 0.5 and 4 h. # signifies receptors that did not have sufficient association (Insignificant cell binding compared to mIgG1 isotype control); therefore internalization could not be accurately calculated. Data is mean ± SD (*n* = 3). Each data point represents mean of a single donor (three technical replicates). Data is mean ± SD (*n *= 3).

Consistent with the literature, CD27 receptor expression is 4x lower on B cells than T cell subsets, whereas CD38 expression is slightly higher on B cells (Figures 4 and 7).^[^ ^78^ ^]^ While the antibody binding differed, the internalization kinetics for both CD27 and CD38 were similar on B cells to T cells (Figure 8), with minimal internalization measured after 30 min and ≈30% internalization after 4 h (Figures 6, 9 and 6, 9).

The common lymphocytic markers, CD44 and CD45, again showed high expression on B cells (MFI 31256 ± 8309 and 53907 ± 5834, respectively) (Figure 7).^[^ ^63^, ^64^ ^]^ However, uptake was even lower than that observed with T cells. <1% internalization was observed for both receptors after 24 h.

Our results highlight the importance of screening and understanding the relationship between mAb binding and cellular uptake of mAb/receptor pairs. As expected, there wasn't a consistent correlation between antibody association and internalization, and we found no clear trends that would allow a prediction of uptake from association (Figure S16, Supporting Information). Some receptors have well‐established uptake pathways, such as CD3 and CD22, which are proposed to be taken up via rapid clathrin‐mediated endocytosis,^[^ ^74^, ^79^, ^80^ ^]^ while CD44 is a classical marker for the CLIC/GEEC pathway,^[^ ^81^, ^82^ ^]^ which has much slower internalization kinetics. While known receptor uptake mechanisms should be considered when choosing therapeutic targets, understanding the effect the mAb is having on the receptor is equally critical for therapeutic outcome. This information can dictate whether a mAb should be employed as an ADC for cargo delivery or engineered as a blocking or ADCC mAb for immune modulation, depending on its internalization behavior.^[^ ^83^, ^84^, ^85^ ^]^ It is also important to note that antibodies that bind to different epitopes on the receptor can exhibit different internalization behavior.^[^ ^35^, ^86^, ^87^ ^]^ For some antibody therapeutics such as antibody‐drug conjugates, the subcellular trafficking that occurs following internalization (e.g., endosomal escape), as well as linker types and payloads can play a major role in activity of these therapeutics.^[^ ^88^ ^]^ However, activity of these conjugates could be improved by selecting for antibodies with more favorable internalization profiles. Furthermore, it is observed that there is significant patient‐to‐patient variability in internalization of key targets for cancer therapy, which may have a significant impact on treatment outcomes. Therefore, ability to rapidly and accurately quantify receptor internalization and mAb uptake has the potential to advance a wide range of therapeutic and diagnostic areas.

Combined, these data demonstrate the capability of the SHIP staple sensor to execute high‐throughput screening of mAbs in complex cell populations, assessing multiple targets across different cell types. We have shown that the SHIP staple sensor system can broadly be used to screen and evaluate mAb cell binding and uptake via target receptors to establish internalization efficiency of diverse functional receptors on subpopulations of lymphocytes.

## 3 Conclusion

Understanding the characteristics of mAb‐receptor interactions is critical for the development of mAb therapies. Screening for the relationship between mAb binding and uptake, and the receptor internalization efficiency, can enable the development of more specific and efficacious mAbs. The one‐pot SHIP staple sensor allows for reproducible and comparable measurement of receptor internalization with mAbs. This adaptable method can be used to garner valuable information on quantifying mAb binding and uptake into the cell and receptor internalization kinetics, which may be used to screen for antigen targets or means to evaluate antibody uptake through a single receptor to select for desirable internalization kinetics. There is a significant benefit when developing therapeutics to distinguish between antibodies that have agonistic effects, promoting receptor internalization and uptake, which could be employed for ADCs,^[^ ^83^ ^]^ or those that remain surface‐bound and used to block receptors at the cell surface or engineered for ADCC activity.^[^ ^84^, ^85^, ^87^ ^]^ Generated immune responses are largely driven by receptors present on immune cells that act to regulate activation, differentiation, and proliferation.^[^ ^89^, ^90^, ^91^ ^]^ Modulating these receptors can aid to either enhance immune responses against cancerogenic antigens, or block responses that promote immune tolerance. However, it is critical to understand the function of the mAb and its effect on these immune receptors to have the desired therapeutic outcome. The staple sensor system is a simple tool for high throughput screening to understand the effect of the mAb on receptor internalization. This information will provide valuable insights in early stages of developing targeting mAbs for nanoparticle functionalization, or for clinical mAb development. More broadly, development of a chemical modification‐free approach to conjugating mAbs to DNA using the DNA/protein staple system could also be expanded to other DNA protein conjugations. This includes screens for the therapeutic efficiency of antibody‐oligonucleotide conjugates made with antisense oligonucleotides or other nucleic acids therapeutics.^[^ ^92^ ^]^

## 4 Experimental Section

### DNA/Protein Staple Expression

The gene encoding for TP1107‐VirD2‐6xHisitidine (VirD2 encodes for amino acids 1–204) was cloned into a bacterial expression pET‐24(+) vector (Novagen). The fusion protein was expressed in BL21(DE3) *E. coli* and purified using immobilized metal affinity chromatography (Cobalt‐NTA). The protein was concentrated using a 50 kDa Amicon centrifugal filter unit and buffer exchanged into 50 mM HEPES buffer. Final protein concentration was determined by Nanodrop at 280 nm.

### Oligonucleotide Design for Sensor

The oligonucleotides used for the staple SHIP sensor were modified to contain the VirD2 DNA‐binding site. The oligonucleotides were designed as a 3‐part system to form the FIP/Quencher pair for the SHIP assay. All ssDNA oligonucleotides were ordered from Integrated DNA Technologies (IDT). 1) 5′ ‐CAA CGG TAT ATA TCC TGC CAG CAT AGT TAC GCT GAA TGA GAT GGA CGC 3′, 2) 5′ Cy5‐ TCA GTT CAG GAC CCT CGG CTG CGT CCA TCT CAT TCA GCG T 5′, 3) 5′ AGC CGA GGG TCC TGA ACT GA‐BHQ2 3′. The oligonucleotide for the click sensor was 4) 5′ Cy5‐ TCA GTT CAG GAC CCT CGG CT ‐NH_3_ 3′. Oligonucleotides were reconstituted in Milli‐Q water to prepare 50 µM stocks. Oligonucleotides 1) and 2) were hybridized in a 1:1 ratio and used for VirD2‐DNA binding. Oligonucleotide 3) is the quencher DNA (QP_C_) and was used for quenching in the SHIP assay for both the staple and click sensors.

### One‐Pot Sensor Bioconjugation Reaction and Gel Analysis

Purified TP1107‐VirD2 protein was reacted with DNA and antibody in a 1:1:1 molar reaction in HEPES buffer (50 mM). First protein and DNA were reacted at 37 °C for 60 min. Antibody was then added for a further 30 min. Reactions were not purified.

Reactions were verified by SDS‐PAGE using a pre‐cast, 12% TGX polyacrylamide gel (Bio‐Rad). 20 µL of the conjugation reactions (and respective controls) were prepared with 5 µL of 1% 2‐mercaptoethanol in SDS‐page loading buffer and heat treated at 94 °C for 3 min prior to loading in the gel well. Samples containing antibody were not denatured (no 2‐mercatoethanol was added nor was the sample heat treated). 5 µL of nondenaturing SDS‐page loading buffer was added to the samples and loaded into the gel wells. The gel was run for 90 min at 120 V in running buffer (25 mM Tris, 250 mM glycine, 0.1% SDS at pH 8.3).

For Western blot, the gel was transferred onto a nitrocellulose membrane using a Bio‐Rad Trans‐Blot Turbo Transfer System (as per the manufacturer's instructions). Anti‐Histidine HRP‐linked antibody was added (1:2000 dilution in blocking buffer) and probed overnight at 4 °C. Chemiluminescence substrate was added as per the manufacturer's instructions, and the membrane was scanned using the Bio‐Rad ChemiDoc Imager (Bio‐Rad) on the chemiluminescence and colorimetric channels. Channel images were merged for the final image.

### Densitometry Calculations of Products in Conjugation Reactions

The percentage of conjugation was determined by densitometric analysis of the Western blot or image of Cy5 gel using ImageJ/FIJI. The total signal (protein or Cy5) of a single lane was determined, and the intensity of the relevant‐sized band was used to calculate the percentage of that product present in the lane and, therefore, the reaction.

### TFR Antibody Conjugation with FIP

TFR (OKT9) antibody, in Sodium Bicarbonate buffer, was reacted with a 10x molar excess of NHS‐DBCO for 2 h at 37 °C. After 2 h, the reaction was passed through a ZEBA column (ThermoScientific) into PBS, to remove unreacted NHS‐DBCO. The concentration of the DBCO‐Antibody was measured by Nanodrop (ThermoScientific). FIP‐azide was then added at a 5x molar excess and incubated overnight at 4 °C. The FIP‐antibody was then purified by microcentrifugation using a 50k Amicon filter to remove free FIP‐azide.

Antibody‐FIP conjugation was measured using Nanodrop, and the Degree of Labelling was calculated by Equation (1),^[^ ^93^ ^]^ below:

(1) $$DOL=\frac{A_{dye}\;\times \in_{protein}\;}{\in_{dye}\left(A_{280}\;-\;CF\;\times \;A_{dye}\right)}$$

where *A_dye_* is the absorbance of the dye at its absorbance maximum (for Cy5 is 649 nm), $\in_{dye}$ is the extinction coefficient of the dye at its absorbance maximum, *A*_280_ is the absorbance of the antibody conjugate at 280 nm, $\;\in_{protein}$ is the extinction coefficient of the antibody at 280 nm, and CF is the empirically obtained correction factor which takes into account the absorbance of the dye at 280 nm. For Cy5‐FIP, the CF is ≈0.55. The calculated DOL is then used to normalise MFI values obtained by flow cytometry. This is achieved by dividing the obtained MFI by the DOL.

### Cell Culture

The C1R and A549 cell lines were maintained at 37 °C and 5% CO_2_ in DMEM with GlutaMAX supplemented with 10% FBS. Cells were used up to passage 30 and routinely tested for mycoplasma contamination.

### Extraction of Primary PBMCs

Healthy donors aged between 18–50 years old in both sexes were recruited voluntarily after the invitation to participate. The ethics is approved by Monash University Human Research Ethics Committee, application ID 37405. Primary PBMCs were isolated from donor blood samples by density gradient medium centrifugation using Ficoll‐Paque media. Briefly, collected blood is diluted with an equal volume of PBS solution and layered on top of Ficoll‐Paque media (Merck, USA) in a 50 mL centrifugation tube. Samples are centrifuged for 30–40 min (400×g) at 18 °C to 20 °C (no brake). After centrifugation, plasma (the upper layer) was removed and discarded to reveal the layer of PBMCs which were collected. PBMCs were then diluted with 3× PBS and centrifuged at 400×g for 10 min (18–20 °C) to remove residual media. This step was then repeated. PBMCs were finally resuspended in RPMI media with 2% FBS for use, or FBS with 10% DMSO for cryo‐preservation of the PBMCs.

### Cell Surface Association Assay (In Vitro)

Cells were seeded into a 96‐well V‐bottom plate at 5 × 10^4^ cells per 50 µL well. Samples were added into separate wells in triplicates. Sensors were added to a final concentration of 25 nM of calculated conjugate product. Cells were incubated with the samples for 60 min at 4 °C. Cells were washed thrice with DMEM with 1% BSA before and resuspended in 50 µL in the same buffer. Cell association of samples was then analyzed by flow cytometry (see Supporting Information).

### Internalization Assay

In vitro, Cells were seeded into a 96‐well plate at a concentration of 5 × 10^4^ cells mL^−1^ (for in vitro cells). Samples were added into separate wells in triplicates to a final concentration of 25 nM of calculated conjugated products from one‐pot reactions or 25 nM of antibody‐FIP (Click sensor). Samples were incubated at 37 °C as per the indicated time points and 4 °C for the longest time point. To stop the reaction cells were cooled to 4 °C. Cells were then washed twice with PBS/1%BSA and resuspended in PBS/1%BSA and half the volume of the well was transferred to a second well. Finally, one half is resuspended in PBS and the other in PBS/BHQ2 (solution of 500 nM quencher DNA). Cells were incubated with BHQ2 for 10 min at 4 °C and analyzed using flow cytometry (see Supporting Information).

### Ex Vivo PBMCs

Staple sensors were generated for each immune receptor antibody (1:1:1 molar ratio of DNA/protein staple: DNA: IgG1 antibody). Staple sensor was added at a final concentration of 10 nM (in 70 µL) to 5 × 10^5^ primary PBMCs in a well. Time points for internalization were taken at 0.5 and 4 h to determine which receptor internalization kinetics for B cell, T cell and monocyte receptors.

Samples were incubated at 37 °C as per the indicated time points and 4 °C for the longest time point. To stop the reaction, cells were cooled to 4 °C. Cells were then washed twice with RPMI /2%FBS and resuspended in RPMI/2%FBS containing the relevant phenotyping panel. Cells were incubated with phenotyping antibodies at 4 °C for 30 min. Cells were then washed twice with RPMI /2%FBS and half the volume of the well was transferred to a second well. One of the two wells is resuspended in in PBS and the other in PBS/BHQ2 (solution of 500 nM quencher DNA). Cells are incubated with BHQ2 for 10 min at 4 °C and analyzed using flow cytometry (see Supporting Information).

Phenotyping antibodies were used to determine PBMC cell populations. For *B cell/ CD4+ T cell/ CD8+ T cell populations*, anti‐Human CD4‐BV510 (BioLegend), anti‐Human CD8‐BV785 (BioLegend), anti‐Human CD19‐BV421 (BioLegend), anti‐Human TCR‐PE (Thermo Fisher), Zombie‐NIR (Cell Live/Dead stain) (BioLegend) were used. When using the anti‐CD19 staple sensor, the CD19‐BV421 phenotyping antibody was substituted for a CD20‐BV421 (BioLegend) phenotyping antibody to determine the B cell population.

### Calculating Internalization

Internalization (%) was calculated via Equation 2 below, where P_n_ is the geometric MFI at time n without the addition of QP_c_. Q_n_ is the geometric MFI at time n after QP_c_ has been added. Q_Eff_ is the quenching efficiency. Quenching efficiency is given by Equation 3 below, where the same annotations apply, but samples are incubated at 4 °C.^[^ ^93^ ^]^

(2) $$\%\;internalised\;\left[37\;C\right]=\left[1-\left(\frac{Pn-Qn}{Pn*\left(1-Qeff\right)}\right)\right]*100$$

(3) $$Quenching\;Efficiency\;ratio\;\;\left[4\;C\right]=\left[1-\left(\frac{Pn}{Qn}\right)\right]$$

### Statistical Analysis

Data are presented as mean ± standard deviation (*mean* ± *SD*) based on the data obtained from at least *n* = 3 independent experiments or well, as indicated in the figure caption. Data which has been normalized is indicated within the figure caption. Statistical tests and significance were determined using GraphPad Prism 9.0 and stated in each figure legend. Tests performed were either a One‐Way or 2‐Way ANOVA (with post‐hoc Dunnett's Test) or unpaired T‐test, as indicated in the figure captions. For all figures and data, p>0.05 is not significant (ns), * = *p *< 0.05, ** = *p* < 0.01, *** = *p* < 0.001 and **** = *p* < 0.0001 (as indicated in captions).

## Conflict of Interest

The authors declare no conflict of interest.

## Supporting information



Supporting Information

## Data Availability

The data that support the findings of this study are available from the authors upon reasonable request.
