# Supplementary material for: A Method for High‐Throughput Screening of Monoclonal Antibody Internalization Using a DNA/Protein Molecular Staple
Source: Small Methods. 2025 Sep 23;9(11):e01399. doi: 10.1002/smtd.202401399 (PMC12641364; doi:10.1002/smtd.202401399)
Supplement: Supplementary file 1 — Supporting Information [file SMTD-9-e01399-s001.docx]

***A Method for High-Throughput Screening of Monoclonal Antibody Internalization using a DNA/Protein Molecular Staple***

Lara M. Mollé, Cameron H. Smyth, Bruna Rossi Herling, Daniel Yuen, Angus P.R. Johnston*

L.M. Mollé, C.H. Smyth, B. Rossi Herling, D. Yuen, A.P.R. Johnston

Drug Delivery, Disposition and Dynamics, Monash Institute of Pharmaceutical Sciences, Monash University, Parkville, Victoria, Australia
E-mail: [angus.johnston@monash.edu](mailto:angus.johnston@monash.edu)

**Supplementary Information**

***Supplementary Methods***

*Protein expression*

*TP1107-mVirD2-6xHisitidine* (amino acid sequence below) protein was expressed in BL21(DE3) *E. coli*. Transformed *E. coli* were grown overnight in LB at 37ºC, 180 rpm. The next day, cultures were transferred into 0.5 L of TB and cultured at 37ºC until OD 1. When reached, IPTG was added to a final concentration of 1 mM, and the temperature was decreased to 30ºC for 16 hours. Cells were harvested by centrifugation (15 min, 4500xg). Cell pellets were resuspended in 10 mL NTA column wash buffer (50 mM NaH_2_PO_4_, 300 mM NaCl, 10 mM Imidazole, pH 8.0). EDTA-free protease-inhibitor (1 tablet of cOmplete, Roche), chicken egg lysozyme (10 µg) and benzonase nuclease (10 µL) was added. Cells were disrupted by homogenisation (25,000 psi) and the lysate was centrifuged (12,000xg, 1 hour). The supernatant was loaded onto a Cobalt-NTA column and washed with two column volumes of NTA column wash buffer. Protein was eluted in NTA elution buffer (50 mM NaH_2_PO_4_, 300 mM NaCl, 250 mM Imidazole, pH 8.0) and concentrated by Amicon centrifugal filter unit (50 kDa cut-off) and buffer exchanged into 50mM HEPES buffer. Final protein concentration was determined by Nanodrop at 280nm.

*TP1107-mVirD2-linker-6xHistidine amino acid sequence*

MGASQVQLVESGGGLVQPGGSLRLSCAASGFTFSDTWMNWVRQAPGKGLYWISAINPDGGNTAYADSVKGRFTISRDNAKNMVYLQMDNLRPEDTAMYYCAKGWVRLPDPDLVRGQGTQVTVSSGGCASPDRAQVIIRIVPGGGTKTLQQIINQLEYLSRKGKLELQRSARHLDIPVPPDQIRELAQSWVTEAGIYDESQSDDDRQQDLTTHIIVSFPAGTDQTAAYEASREWAAEMFGSGYGGGRYNYLTAYHVDRDHPHLHVVVNRRELLGHGWLKISRRHPQLNYDGLRKKMAEISLRHGIVLDATSRAERGIAERPITYAEHRRLERMQPWTGAHIVMVDAYKPTKTGENLYFQSHHHHHH*

*Flow Cytometry*

Flow cytometry was performed with a Stratedigm S1000EXI flow cytometer (Stratedigm, California, USA). For analysis of Cy5 signal, a 642 nm excitation emission was collected between 661 – 691 nm across all samples. FCS2.0 files were exported from CellCapTure Analysis Software (Stratedigm, California, USA) and gated by forward and side scatter in FlowJo (version 10.10.0, Tree Star, Oregon, USA) before further analysis.

*Monoclonal Antibodies for Staple Sensors*

All mAbs used in this study were purified mouse monoclonal IgG1 anti-human antibodies. TFR antibody (clone OKT9) purchased from WEHI Antibody Facility. mIgG1 isotype control (clone P3.6.2.8.1) (Invitrogen). CD2 (clone RPA-2.10) (Invitrogen). CD3 (clone UCHT1) (Invitrogen). CD4 (clone L200) (BD Pharmingen). CD5 (clone UCHT2) (Invitrogen). CD7 (clone 124-1D1) (Invitrogen). CD8a (clone HIT8a) (Invitrogen). CD14 (clone 61D3) (Invitrogen). CD19 (clone HIB19) (Invitrogen). CD20 (clone MEM-97) (Invitrogen). CD22 (clone 4KB128) (Invitrogen). CD27 (clone O323) (Invitrogen). CD38 (clone HIT2) (Invitrogen). CD44 (clone SFF-2) (Invitrogen). CD45 (clone HI30) (Invitrogen).

*Human PBMC collection and purification*

Healthy donors aged between 18 – 50 years old of both sexes were voluntarily recruited following invitation to participate. The ethics is approved by Monash University Human Research Ethics Committee, application ID 37405. 10-30 mL of human blood was collected and diluted with PBS before carefully layered on Ficoll-Paque PLUS density gradient media with 1:1 v/v. The layer of PBMCs collected after 400g, 40 mins spin and washed with prewarmed RPMI media twice. Isolated PBMCs were other used for experiments or frozen in cell media at -80°C and thawed for experimental use. Thawed PBMCs were recovered in prewarmed media and incubated for 2 hours at 37 ºC to recover cells prior to experimental use.

*
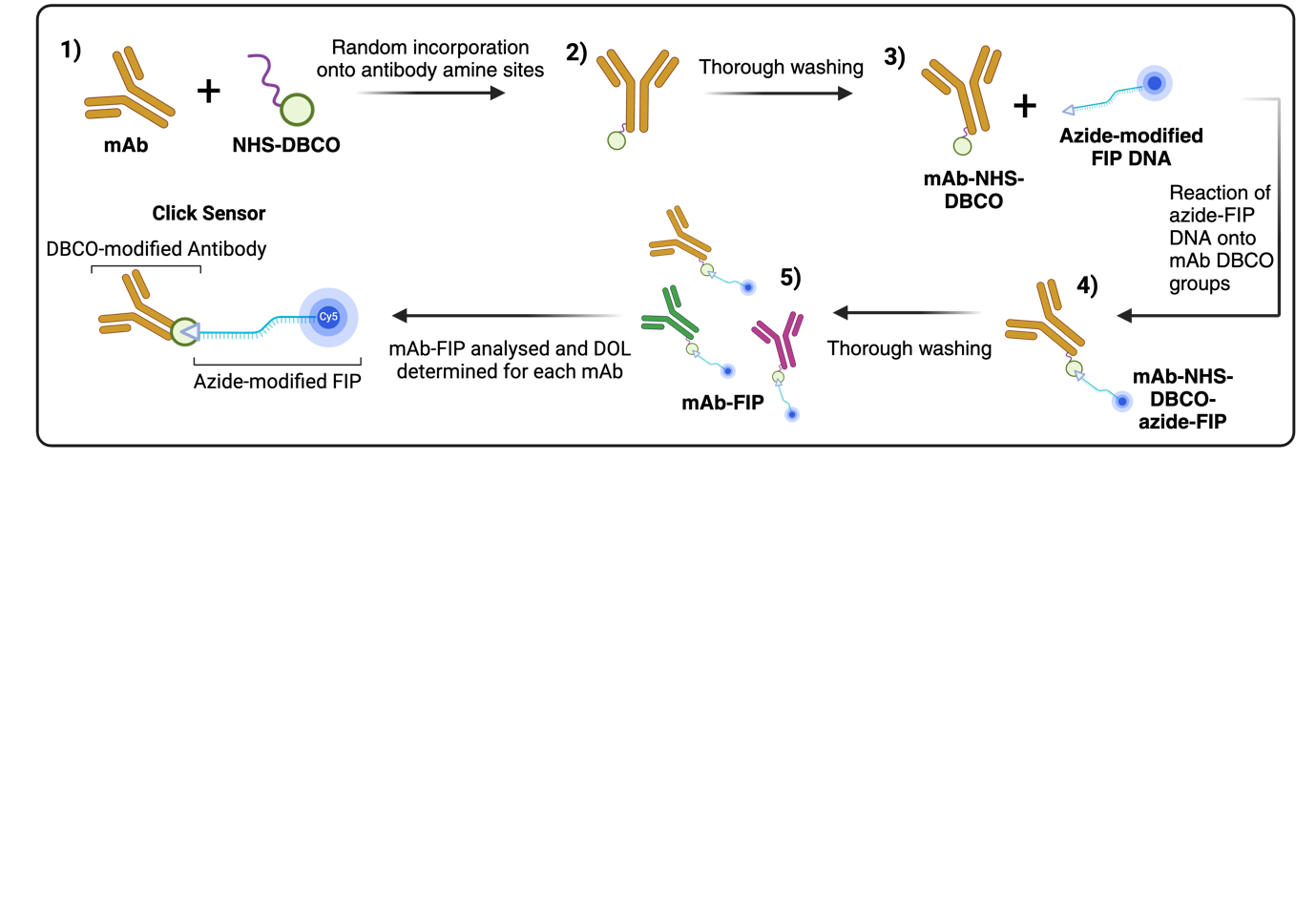
*

***Figure S1.*** *The click SHIP sensor requires a multi-step time consuming process which needs to be repeated for each antibody of interest. This involves 1) reacting NHS-DBCO onto random amines in the mAb in an overnight reaction step. 2) This is then followed by thorough washing to remove unreacted NHS-DBCO and organic solvent. 3) The DBCO-mAb is then reacted with azide-FIP DNA overnight and 4) the unreacted azide-FIP is then removed through multiple wash steps. 5) This process must be repeated for each mAb of interest and the degree of labelling (DOL) calculations must be performed for each labelled antibody and accounted for during analysis. Figure generated with BioRender.com*

*
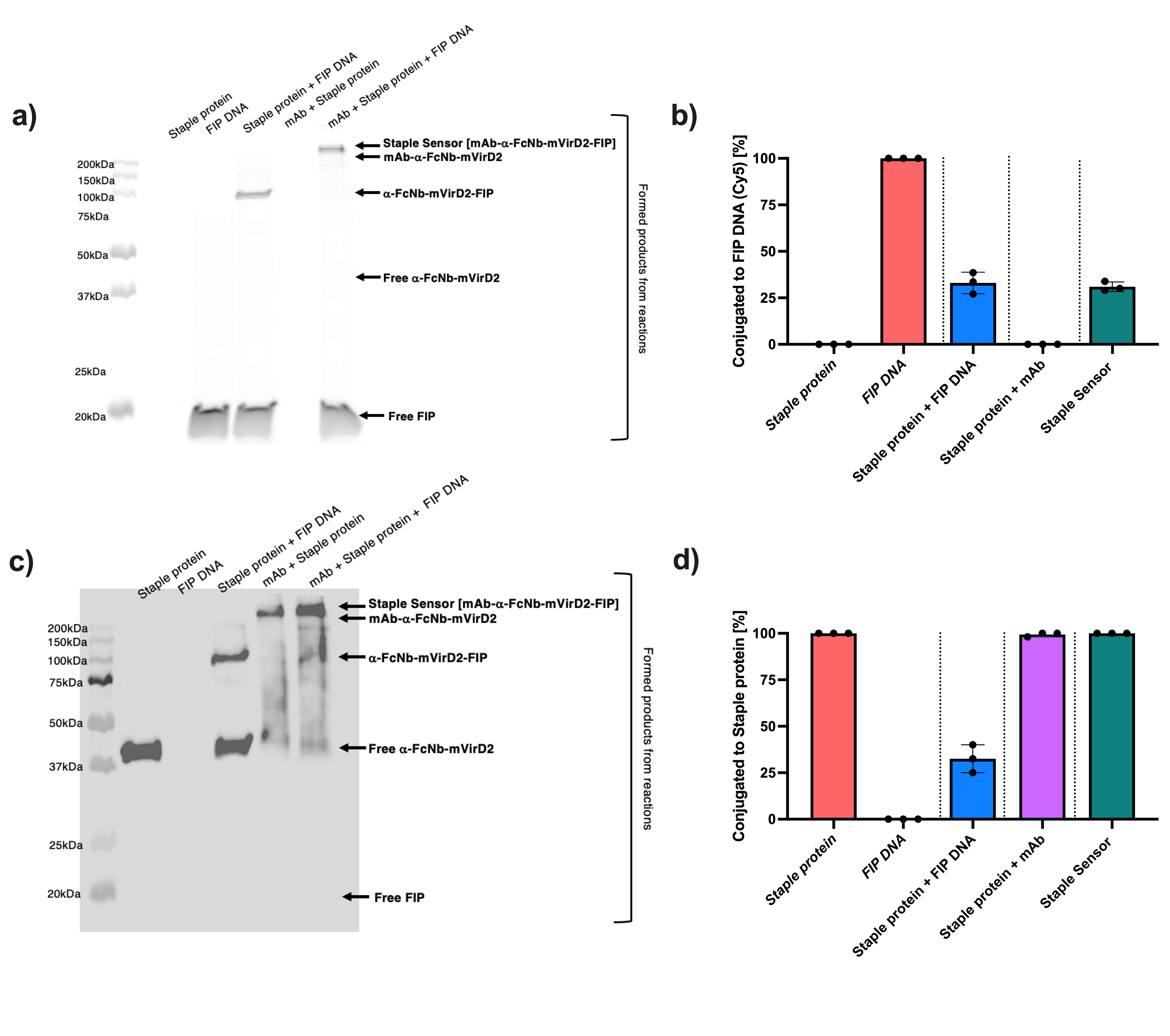
*

***Figure S2.*** *Formation of Staple sensor in one-pot reaction using an anti-TFR antibody (OKT9). Binding of Staple protein to DNA and mAb was assessed by SDS-PAGE. A) Fluorescence image of gel at 642nm to detect Cy5 labelled DNA B) Western Blot of the gel using an anti-Histidine tag antibody against the staple protein. C) Densitometry quantification of anti-histidine tag western blot was used to determine % conjugated Tp1107-VirD2 to either FIP or mAb. Densitometric analysis of the Cy5 image of SDS-PAGE gels and corresponding Western blots was used to determine ratios formed staple sensor, staple protein binding to DNA and staple protein binding to mAbs as depicted in Figure 3.*

***
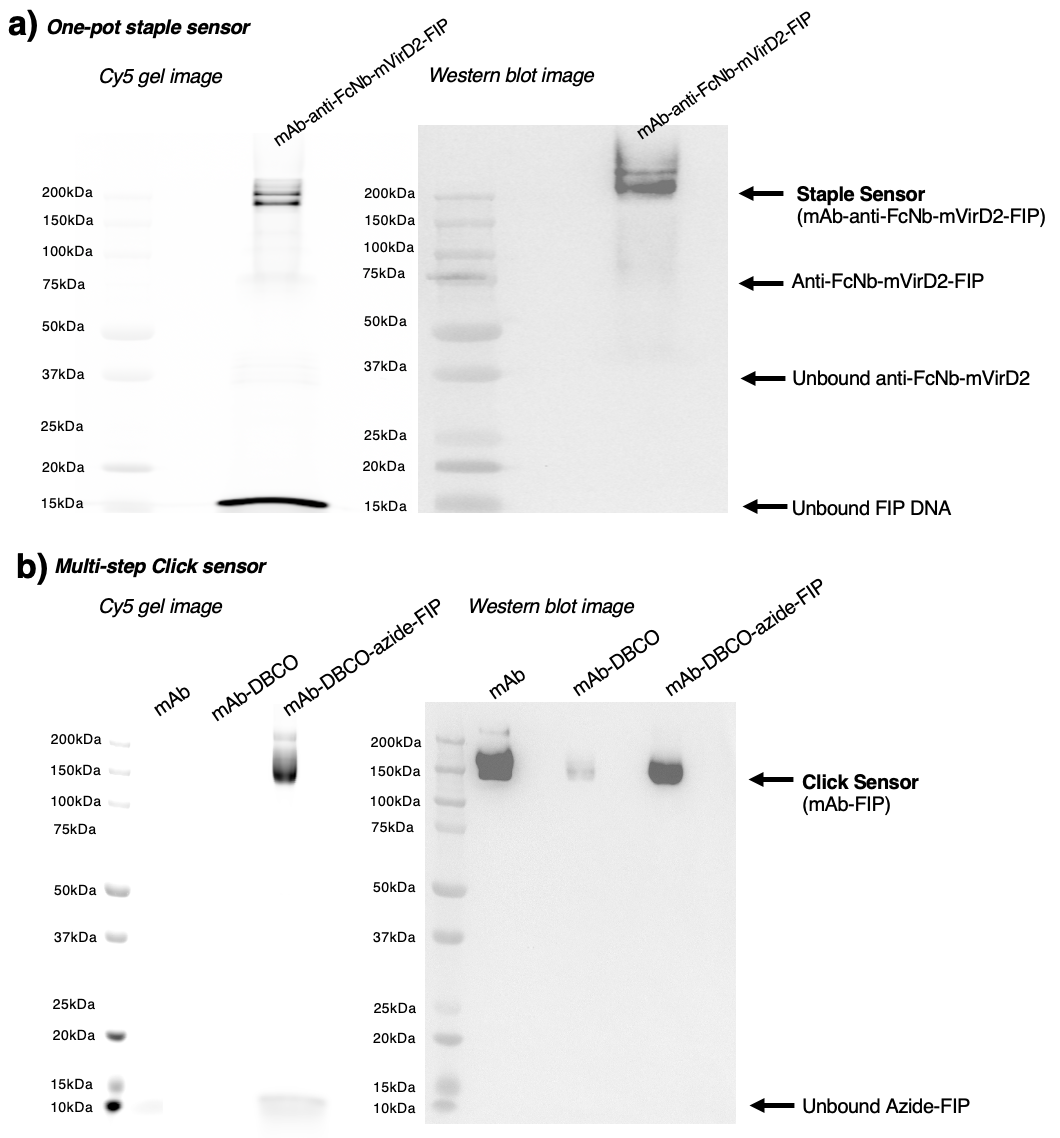
***

***Figure S3.*** *Formation of Staple and Click sensors with an anti-TFR antibody (OKT9). A) The Staple sensor is formed in a one-pot reaction by reacting Staple protein, FIP DNA and antibody (OKT9). Staple sensor formation was confirmed by SDS-PAGE followed by Cy5 imaging of the gel and western blot analysis using an anti-Histidine tag antibody against the staple protein. B) The Click sensor is formed in a multi-step process. Antibody is reacted with NHS-DBCO followed by purification to remove excess DBCO. Then, Azide-FIP is added in excess to form the sensor. Click sensor formation is confirmed by Cy5 imaging of the SDS-PAGE gel and Western blotting using an HRP-linked anti-mouse antibody against the OKT9 antibody.*

*
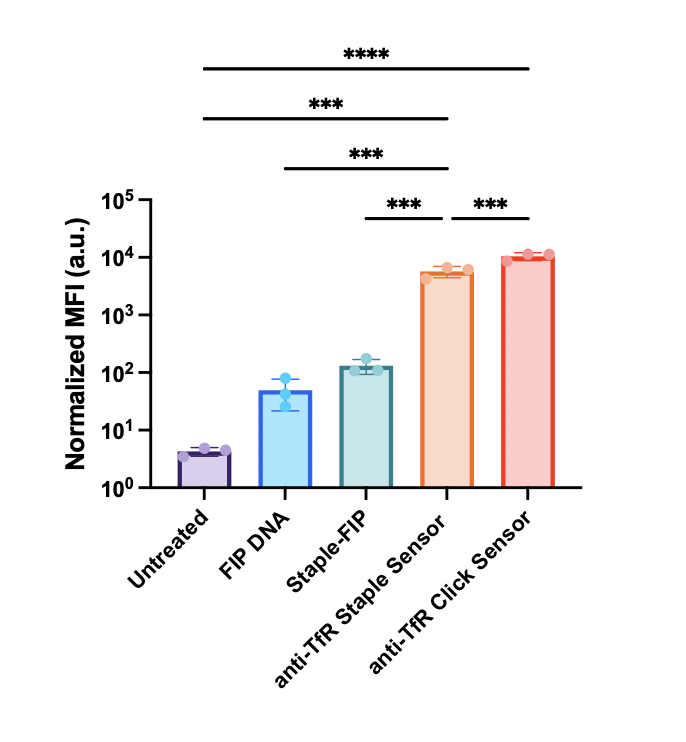
*

***Figure S4.*** *Log scale depiction of the* *staple sensor binding to TfR on cultured B cells. One-pot anti-TfR staple sensor TfR was incubated with B-lymphoblastoid (C1R) cells for 1 hour at 37ºC. MFI (Cy5) was measured by flow cytometry. Click sensor MFI was corrected for the DOL (0.6). Ordinary One-Way ANOVA, p>0.05 is not significant and is not indicated on the graphs. * = p<0.05, ** = p<0.01, *** = p<0.001 and **** = p<0.0001, mean* ± *SD (n=3). Each data point represents the mean of one experiment (3 technical replicates).*

*
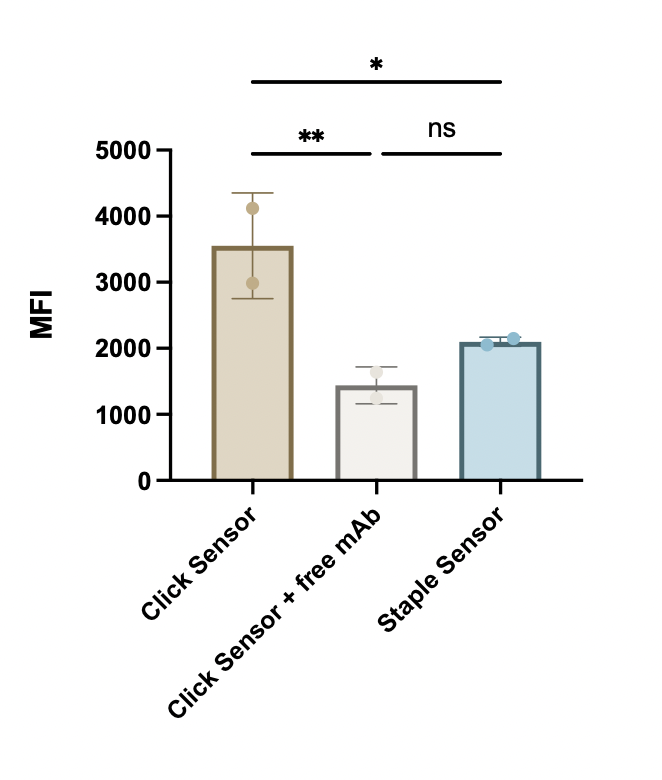
*

***Figure S5.*** *Unlabelled antibody added to the click sensor reduces MFI of cell binding, reflecting the one-pot staple sensor. Free mAb was added to the click sensor to the same final concentration as is present in the one-pot staple sensor (50nM unlabelled antibody to 25nM labelled sensor). 25nM of Staple sensor was added. C1R cells were incubated with click sensor, click sensor + free mAb or staple sensor for 60 minutes. MFI was measured by flow cytometry. Statistical analysis was done using an Ordinary One-Way ANOVA where p>0.05 is not significant (ns) and p<0.05 is given by *. Data points are mean of single experiment (three technical replicates). Data is mean ± SD (n=2).*

*
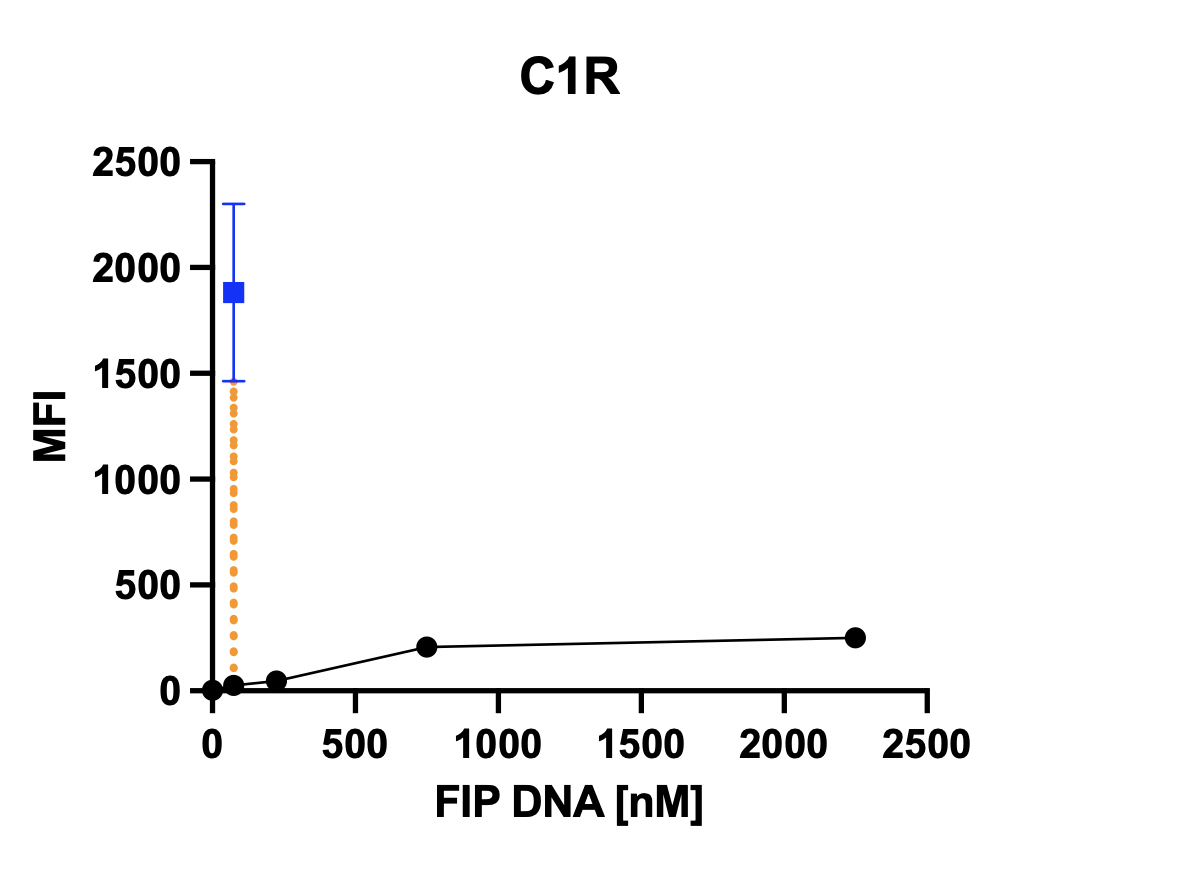
*

***Figure S6.*** *Cell association of free FIP DNA up to 30x concentration of that in the one-pot Staple sensor shows no relevant association with cells. FIP DNA was incubated with C1R cells, at increasing concentrations compared to that used to construct the one-pot Staple sensor: 75nM (1x), 225nM (3x), 750nM (10x) and 2250nM (30x). Association with cells was analysed by flow cytometry at 649 nm to detect Cy5-FIP. Blue data point indicates the targeted TfR staple sensor made in a one pot reaction with 75nM DNA, data taken from Figure 2 (mean ± SD). Dotted orange line indicates concentration of FIP DNA formed into staple sensor. Data is mean ± SD (n=3).*

*
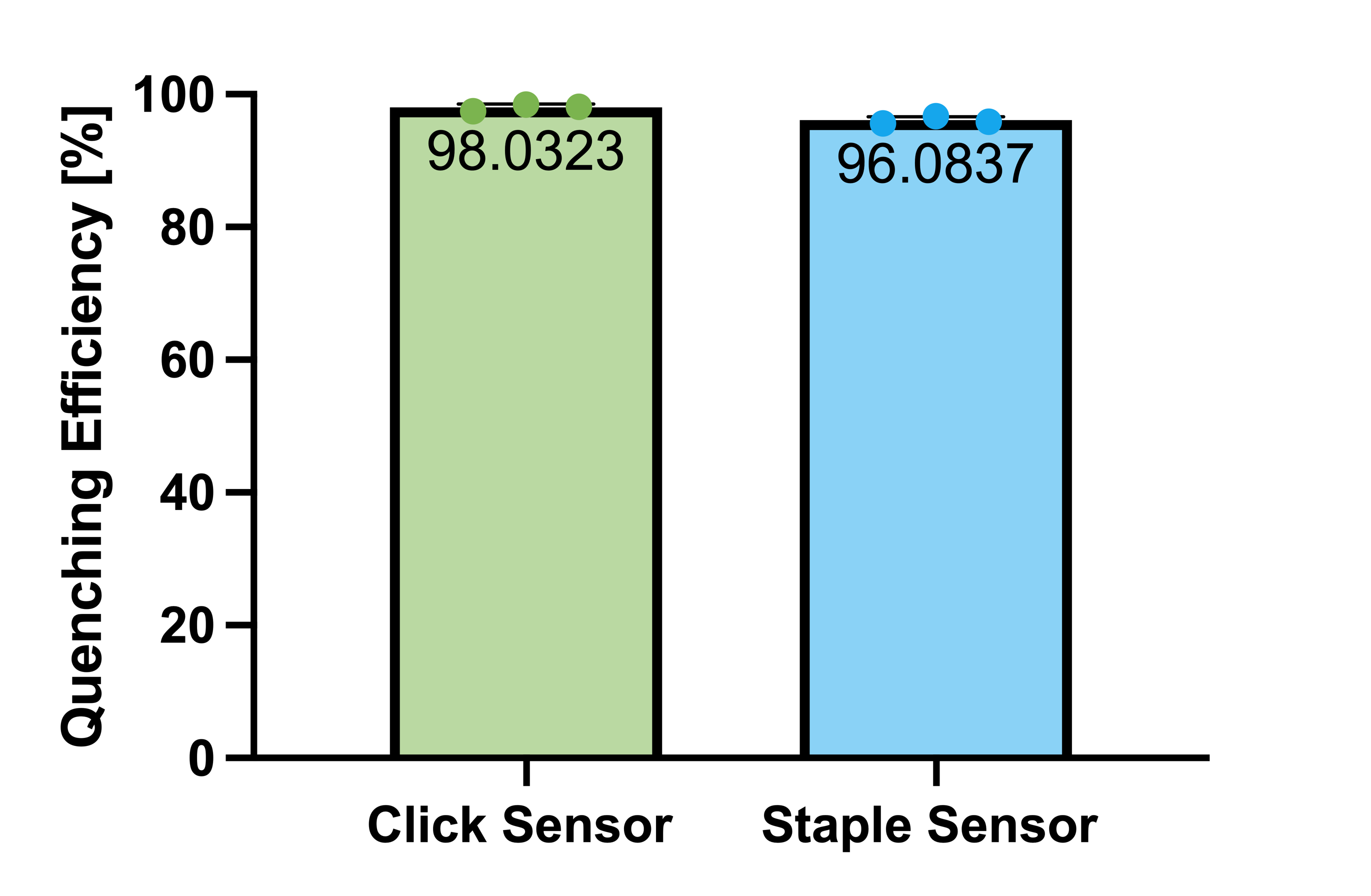
*

***Figure S7.*** *Quenching efficiency of staple and click sensors with 3’ BHQ2 in C1R cells after 4h incubation at 4°C. Average quenching efficiency of three replicate experiments. Data is mean ± SD (n=3).*

*
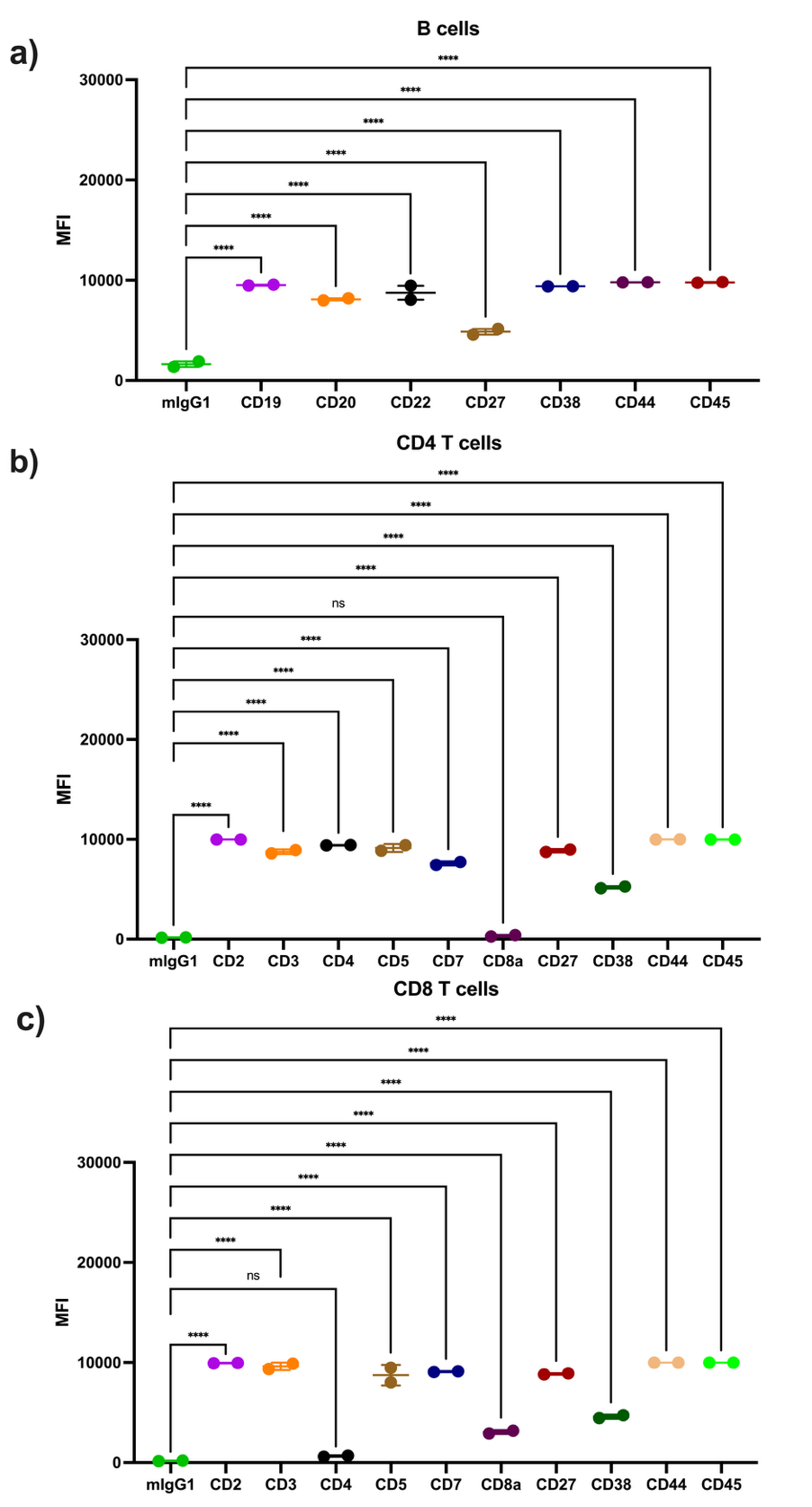
*

***Figure S8.*** *Validation of monoclonal antibodies used for screening internalisation in PBMCs. All mAbs tested at 0.5 ug/mL on cells and detected using an anti-mouse (AF649) secondary antibody. PBMC cell populations were deciphered using phenotyping (Live and Dead dye, antiTCR, antiCD19, antiCD4, antiCD8a and antiCD14) into a) B cells, b) CD4+ T cells and c) CD8+ T cells. Statistical analysis was done using an Ordinary One-Way ANOVA where p>0.05 is not significant (ns) and p<0.0001 is given by ****. Data is mean ± SD (n=2).*

*
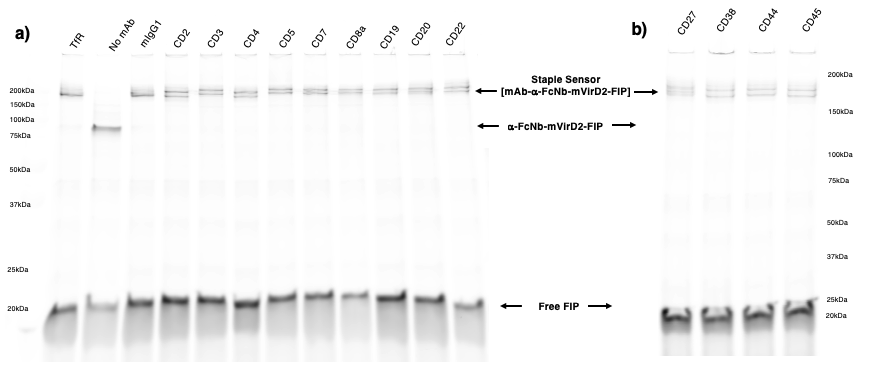
*

*
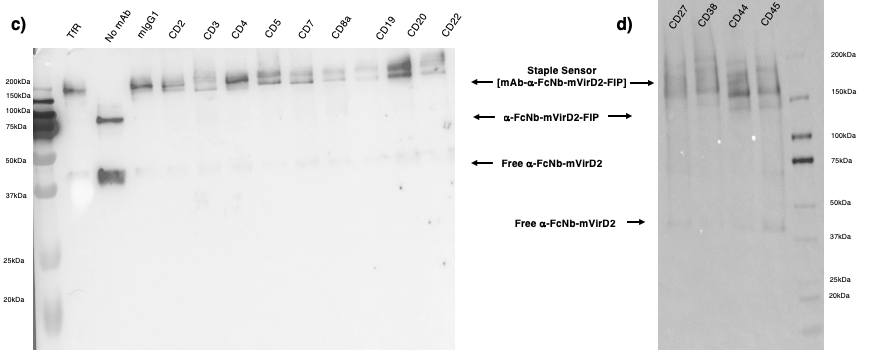
*

***Figure S9.*** *Validation of anti-FcNb-mVirD2 (staple protein) binding to DNA and validated mIgG1 monoclonal antibodies. Staple protein, DNA and mAb were reacted in a 1:1:1 molar ratio and analysed by non-denaturing SDS-PAGE. Fluorescent images (a) and b)) of the gel analysis were used to detect binding of FIP-DNA (Cy5 labelled). Immunoblotting (c) and d)) using an anti-Histidine tag-HRP antibody (against the staple protein) was used for protein detection. Two gels were used to assess binding to all mAbs. Gel 1 is the left hand column and Gel 2 is the right hand column.*

*
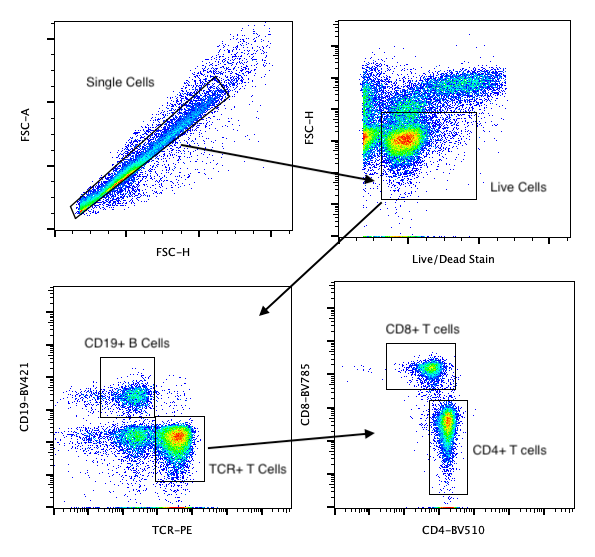
*

***Figure S10.*** *Gating strategy of identifying subpopulation in purified human PBMC. Purified human PBMC were stained with an antibody cocktail (Live and Dead dye, antiTCR, antiCD19, antiCD4, antiCD8a). Lymphocytes were selected first then subphenotyped into different groups as shown in the figure.*

**

***Figure S11.*** *mAb cell binding/ association correlates to relative expression levels of receptors after 0.5 hours. Association of each mAb with each receptor was measured using the mAb-staple sensor. Association in a) CD4+ T cells, b) CD8+ T cells and c) B cells was measured. Cy5 fluorescence was analysed by flow cytometry at 649 nm to detect Cy5-FIP. Statistical analysis was performed using 2-way ANOVA with Dunnett’s test. Each mAb was compared to the isotype (mIgG1) control. p>0.05 is not significant (ns), * indicates p<0.05, ** indicates p<0.01, *** indicates p<0.001 and **** indicates p<0.0001. Significance shown is representative of the mean calculated p value of the donors. Each symbol is representative of a single donor (with technical replicates, Error ± SD). Data is n=3.*

**

***Figure S12.*** *mAb uptake at 0.5 hours is low. Uptake was measured in a) CD4+ T cells, b) CD8+ T cells and c) B cells after quenching. Cy5 fluorescence was analysed by flow cytometry at 649 nm to detect Cy5-FIP. Statistical analysis was performed using 2-way ANOVA with Dunnett’s test. Each mAb was compared to the isotype (mIgG1) control. p>0.05 is not significant (ns), * indicates p<0.05, ** indicates p<0.01, *** indicates p<0.001 and **** indicates p<0.0001. Significance shown is representative of the mean calculated p value of the donors. Each symbol is representative of a single donor (with technical replicates, Error ± SD). Data is n=3.*

**

***Figure S13.*** *Association of a library of antibodies to human CD8+ T cells assessed using staple sensor after 4 hours. Cy5 fluorescence was measured by flow cytometry. Statistical analysis was performed using 2-way ANOVA with Dunnett’s test, n=3. Each mAb staple sensor was compared to the isotype (mIgG1) control. p>0.05 = not significant (ns), * = p<0.05, ** = p<0.01, *** = p<0.001 and **** = p<0.0001. Significance shown is representative of the mean calculated p value of the donors. Each symbol is representative of a single donor (with technical replicates).*

**

***Figure S14.*** *mAb uptake into CD8+ T cells after 4 hour incubation. The MFI of the mAb-staple sensor through each receptor was measured following the addition of quencher DNA (500nM). Cy5 fluorescence was analyzed by flow cytometry. Statistical analysis was performed using 2-way ANOVA with Dunnett’s test, n=3. Each mAb staple sensor was compared to the isotype (mIgG1) control. p>0.05 = not significant (ns), * = p<0.05, ** = p<0.01, *** = p<0.001 and **** = p<0.0001. Significance shown is representative of the mean calculated p value of the donors. Each symbol is representative of a single donor (with technical replicates).*

*
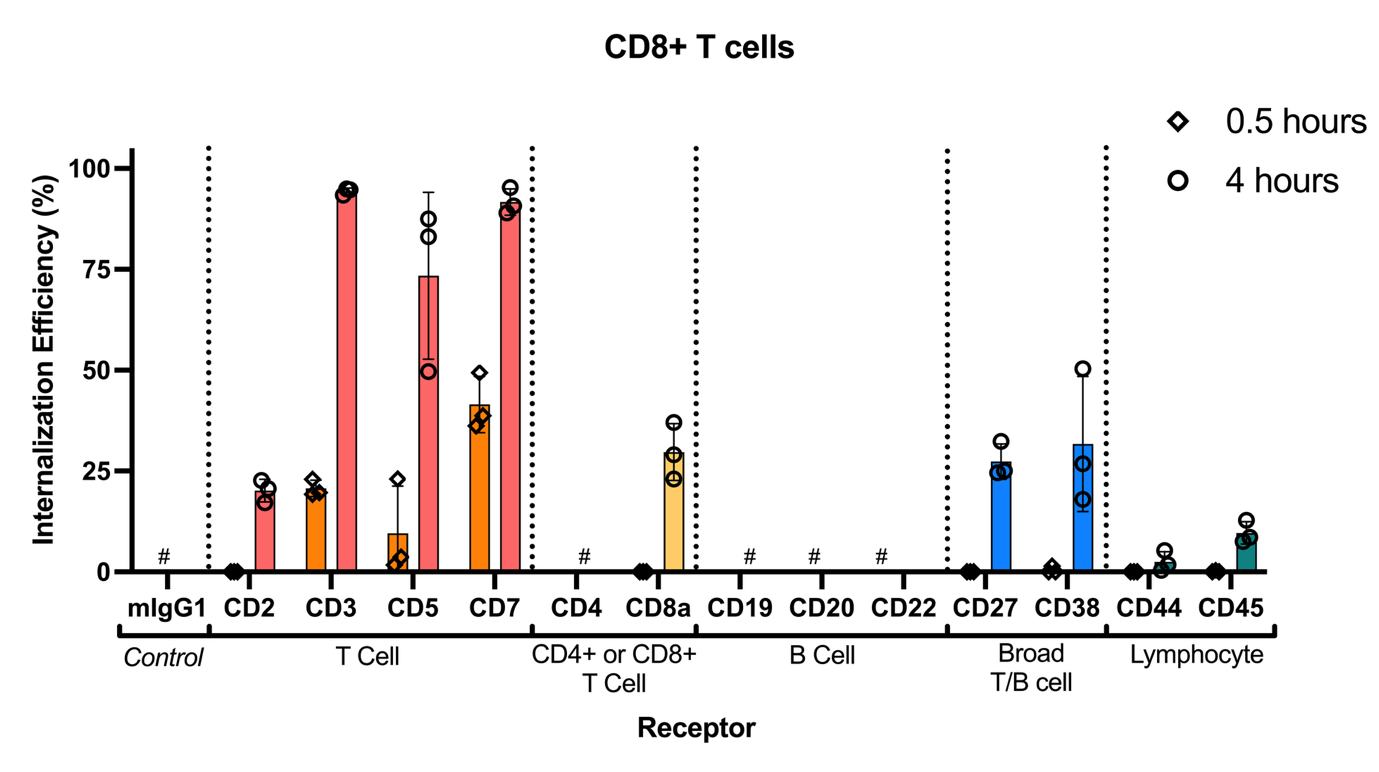
*

***Figure S15.*** *Internalization efficiency increases over time for CD8+ T cell receptors. Internalization efficiency (%) was calculated for each receptor relevant to CD8+ T cells at 0.5 and 4 hours. # signifies receptors that did not have sufficient association compared to mIgG1 isotype control for internalization to be calculated. Each data point represents mean of a single donor (three technical replicates). Data is mean* ± *SD (n=3).*

******

***Figure S16.*** *Correlation analysis of association versus uptake for each receptor and cell type after 4 hours. The mean association of all donors was plotted against the mean uptake for all donors. Red indicates CD4+ T cells, blue indicates CD8+ T cells and green indicates B cells.*
